# The pharmacology and mechanisms of platycodin D, an active triterpenoid saponin from *Platycodon grandiflorus*

**DOI:** 10.3389/fphar.2023.1148853

**Published:** 2023-04-06

**Authors:** Long Xie, Yu-Xin Zhao, Yu Zheng, Xiao-Fang Li

**Affiliations:** State Key Laboratory of Southwestern Chinese Medicine Resources, School of Pharmacy, Chengdu University of Traditional Chinese Medicine, Chengdu, China

**Keywords:** platycodin D, mechanisms, pharmacokinetics, pharmacology, plant

## Abstract

Chinese doctors widely prescribed *Platycodon grandiflorus* A. DC. (PG) to treat lung carbuncles in ancient China. Modern clinical experiences have demonstrated that PG plays a crucial role in treating chronic pharyngitis, plum pneumonia, pneumoconiosis, acute and chronic laryngitis, and so forth. Additionally, PG is a food with a long history in China, Japan, and Korea. Furthermore, Platycodin D (PLD), an oleanane-type triterpenoid saponin, is one of the active substances in PG. PLD has been revealed to have anti-inflammatory, anti-viral, anti-oxidation, anti-obesity, anticoagulant, spermicidal, anti-tumor etc., activities. And the mechanism of the effects draws lots of attention, with various signaling pathways involved in these processes. Additionally, research on PLD’s pharmacokinetics and extraction processes is under study. The bioavailability of PLD could be improved by being prescribed with *Glycyrrhiza uralensis* Fisch. or by creating a new dosage form. PLD has been recently considered to have the potential to be a solubilizer or an immunologic adjuvant. Meanwhile, PLD was discovered to have hemolytic activity correlated. PLD has broad application prospects and reveals practical pharmacological activities in pre-clinical research. The authors believe that these activities of PLD contribute to the efficacy of PG. What is apparent is that the clinical translation of PLD still has a long way to go. With the help of modern technology, the scope of clinical applications of PLD is probable to be expanded from traditional applications to new fields.

## Introduction

*Platycodon grandiflorus* A. DC. (PG, according to Flora of China) is a food with a long history in East Asia. It is termed Jiegeng in China, Doraji in North Korea, and Kikyo in Japan (Zhang et al., 2015a). PG holds a vital place in their dining culture. Its roots, tender leaves, and other parts are of great edible value. For instance, the roots of PG are often eaten as a side dish in daily meals in Korea (Kim et al., 2021). Meanwhile, PG is a widely used medicine dating back to the Han Dynasty. And most doctors agree that PG has therapeutic effects on respiratory diseases with the development of Chinese medical theory (Yin and Fang, 2021). PG is used in many well-known traditional Chinese medicine prescriptions, such as Jiegeng decoction (Mao et al., 2017), Yinqiao powder (Lin et al., 2021), and Sangju decoction (Xu and Zhang, 2020). Moreover, PG is often prescribed to manage cough with phlegm, chest congestion, sore throat, and spitting pus (Yang et al., 2020a; Committee, 2020) in modern times. Furthermore, there is a growing awareness of the health benefits of PG, which is receiving increasing attention due to its effectiveness and high safety profile. From an economic point of view, the great demand for PG can bring economic benefits.

**FIGURE 1:**
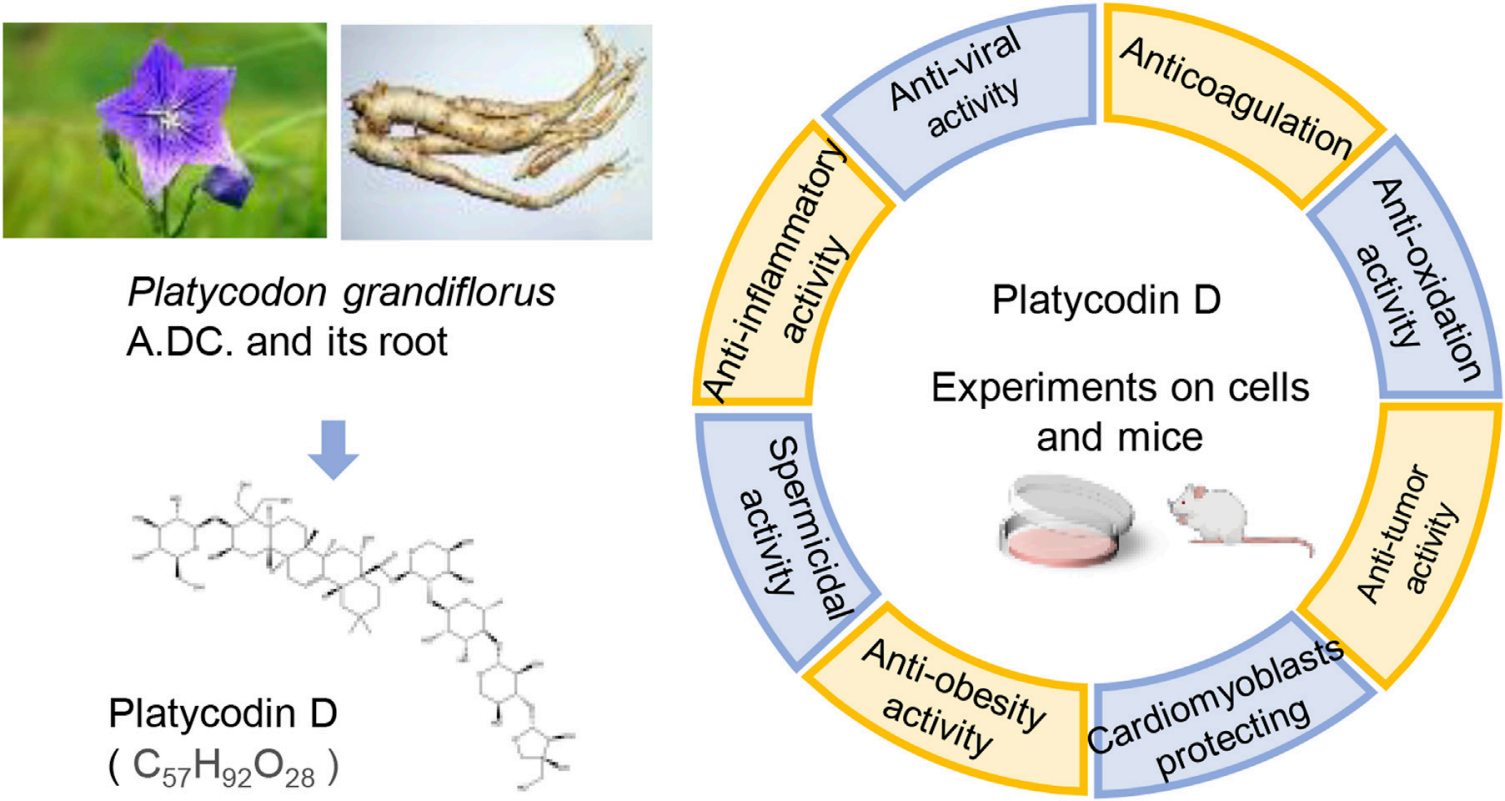
The pharmacological activities of PLD, one of the active constituents of PG. (The left picture of PG, photographed by 663 highland on 3 August 2013, is under licensed by CC BY-SA 3.0. The right picture of PG root, photographed by Asfreeas on 9 November 2010, is under licensed by CC BY 3.0).

Modern research has revealed that PG primarily contains triterpene saponins, flavonoids, phenolic acids, and other substances. Among them, Platycodin D (PLD), an oleanane-type triterpenoid saponin, is one of the main active substances in PD. Triterpenoid saponins have been attracting a wide range of interest from researchers, like oleanane-type saponins. Oleanolic acid has been proven to boost memory (Jeon et al., 2017), trigger cancer cell apoptosis (Jo et al., 2020), as well as weaken migration and invasion (Wang et al., 2019a). Owing to the structural similarity, PLD also possesses similar pharmacological effects. More significantly, the Chinese Pharmacopoeia stipulates that the content of PLD in PG should not be less than 0.10% (Committee, 2020). This provision reflects the medicinal value of PLD to some extent. Combining the above two points, the authors concluded that the pharmacological effects of PLD are important for the efficacy of PG. Consequently, the pharmacological activities of PLD have been reviewed in this paper to facilitate studies on PLD (Figure 1).

## Traditional uses of PG

In China, PG was first recorded in the *Shennong Bencao Jing*, which is said to cure chest pains, bloating, and intestinal tinnitus. The disease with pain in the chest, dry throat, smelly sputum, and spitting pus is referred to as lung carbuncle in *Jingui Yaolue* (written by Zhang Zhongjing, the Han Dynasty). And the Jiegeng decoction in that book is a famous prescription for dealing with lung carbuncle (equivalent to lung abscess nowadays). Afterward, there are numerous records of PG in China. In the Tang Dynasty, it was considered capable of healing sore throats and facilitating digestion (*Xinxiu Bencao* and *Qianjin Yifang*). Moreover, in the Ming Dynasty, it was applied to clear lung heat, open the nasal passages, and detoxify the body and detoxify (*Bencao Mengquan*). In the Qing Dynasty, PG was regarded to eliminate stabbing pains in the hypochondrium, treat sore throats, clear lung heat, and eliminate carbuncles (*Bencao Xinbian*). Meanwhile, doctors’ prescriptions mirrored the significance of PG in managing diseases, especially canker sores (Table 1).

**TABLE 1:** Prescriptions including PG in China’s ancient monographs.

Name of prescription	Prescription	Indications	Author	Monograph/reference (in pinyin)	Dynasty
Jiegeng decoction	PG, *Glycyrrhiza uralensis* Fisch.	Lung carbuncle	Zhongjing Zhang	Jingui Yaolue	Han
Bai powder	PG, *Croton tiglium* L., *Fritillaria thunbergii* Miq.	Lung carbuncle	Zhongjing Zhang	Shanghan Lun	Han
Wine Mixing Xi Gan powder	*Panax ginseng* C.A.Mey., *Rheum palmatum* L., PG, *Anemarrhena asphodeloides* Bunge, *Natrii Sulfas*, *Gardenia jasminoides* J. Ellis, *Scutellaria baicalensis* Georgi.	Eye pain, especially in the first half of the night	Simiao Sun	Yinhai Jingwei	Tang
Jiegeng decoction	PG, *Glycyrrhiza uralensis* Fisch.	Lung carbuncle	Simiao Sun	Beiji Qianjin Yaofang	Tang
Jiegeng Bai powder	PG, *Croton tiglium* L., *Fritillaria thunbergii* Miq.	Lung carbuncle	Xi Wang	Waitai Miyao	Tang
Baidu Liuqi decoction	*Panax ginseng* C.A.Mey., *Pueraria lobata* (Willd.) Ohwi, *Citrus × aurantium* L., PG, *Glycyrrhiza uralensis* Fisch., *Bupleurum chinense* DC., *Saposhnikovia divaricat*a (Turcz.) Schischketc.	Carbuncles on top of the head	Hanqing Dou	Chuangyang Jingyan Quanshu	Jin
Jiegeng Zhiqiao decoction	PG, *Citrus × aurantium* L.	Swelling of the chest and flank	Gong Zhu	Leizheng Huoren Shu	Song
Jiegeng decoction	*Pinellia ternata* (Thunb.) Makino, PG, *Citrus × aurantium* L. or *Citrus sinensis* (L.) Osbeck.	Swelling of the chest and flank, bloating, upset, nausea	Shiwen Chen et al	Taiping Huimin Hejiju Fang	Song
Renshen Baidu powder	*Panax ginseng* C.A.Mey., *Poria cocos* (Schw.)Wolf, *Glycyrrhiza uralensis* Fisch., *Kitagawia praeruptora* (Dunn) Pimenov, Angelica pubescens Maxim., PG, *Bupleurum chinense* DC., *Citrus × aurantium* L.	Headache, fever, aversion to cold, body aches, cough, stuffy nose	Shiwen Chen et al	Taiping Huimin Hejiju Fang	Song
Jiegeng Zhiqiao decoction	*Citrus × aurantium* L., PG, *Pinellia ternata* (Thunb.) Makino, *Scutellaria baicalensis* Georgi, *Trichosanthes kirilowii* Maxim., *Coptis chinensis* Franch.	Chest pain	Yilin Wei	Shiyi Dexiao Fang	Yuan
Chuanxiong Shigao decoction	Gypsum, PG, *Ligusticum striatum* DC., *Paeonia lactiflora* Pall., *Gardenia jasminoides* J. Ellis, *Panax ginseng* C.A.Mey., *Atractylodes macrocephala Koidz*., etc.	Dizziness and pain in the head, dry throat, and polydipsia	Su Dong	Qixiao Liangfang	Ming
Banxia decoction	*Pinellia ternata* (Thunb.) Makino, *Ophiopogon japonicus* (Thunb.) Ker Gawl., *Poria cocos* (Schw.)Wolf, *Atractylodes macrocephala Koidz*., PG, *Citrus × aurantium* L., Peucedanum praeruptorum Dunn, Magnolia officinalis Rehder & E. H. Wilson, etc.	Bloating, vomiting, and no appetite	Su Dong	Qixiao Liangfang	Ming
Jiegeng Xingren decoction	PG, the seeds of *Prunus armeniaca* L., *Glycyrrhiza uralensis* Fisch., *Lonicera japonica* Thunb., *Ophiopogon japonicus* (Thunb.) Ker Gawl., etc.	Cough and vomit pus, blood in sputum, dull pain in the chest	Jingyue Zhang	Jingyue Quanshu	Ming
Yin Qiao powder	*Forsythia suspensa* Vahl, *Lonicera japonica* Thunb., PG, *Mentha canadensis* L., *Glycyrrhiza uralensis* Fisch., *Nepeta tenuifolia* Benth., etc.	Fever without sweat, headache, thirst, or sore throat	Jutong Wu	Wenbing Tiaobian	Qing
Sang Ju decoction	*Prunus armeniaca* L., *Forsythia suspensa* Vahl, *Mentha canadensis* L., *Morus alba* L., PG, *Glycyrrhiza uralensis* Fisch., *Phragmites communis* Trin.	Cough with thick phlegm but cannot cough up	Jutong Wu	Wenbing Tiaobian	Qing
Zhisou powder	PG, *Glycyrrhiza uralensis* Fisch., *Cynanchum stauntonii* (Decne.) Schltr. ex H.Lév., *Citrus × aurantium* L., *Stemona sessilifolia* (Miq.) Miq., *Aster tataricus* L. f.	Cough, sore throat but cannot cough up phlegm, slight fever, and afraid of the wind	Guopeng Cheng	Yixue Xinwu	Qing
Jiawei Jiegeng decoction	PG, *Bletilla striata* Rchb.f., *Citrus × aurantium* L., *Descurainia sophia* (L.) Webb ex Prantl, *Glycyrrhiza uralensis* Fisch., *Fritillaria thunbergii* Miq., Coix lacryma-jobi L., *Lonicera japonica* Thunb.	Lung carbuncle	Guopeng Cheng	Yixue Xinwu	Qing
Anfei Jiegeng decoction	*Prunus armeniaca* L., *Trichosanthes kirilowii* Maxim., *Citrus × aurantium* L., PG, *Angelica sinensis* (Oliv.) Diels, *Astragalus mongholicus* Bunge, *Anemarrhena asphodeloides* Bunge, *Fritillaria cirrhosa* D.Don, etc.	Lung carbuncle	Peiqin Lin	Leizheng Zhicai	Qing

In the past 30 years, PG has gained a significant part in healing diseases of the respiratory system. It was used to treat chronic pharyngitis (Wang, 2011), plum pneumonia (Wang, 2010), pneumoconiosis (Tian et al., 2007), acute and chronic laryngitis (Huang, 1993; Deng, 1998), and children’s cough (Liu and Wen, 1995) in the clinic. In the Chinese Pharmacopoeia, many preparations prescriptions (Chinese medicine) are included, such as Chuanbei Zhike Lu, Xiao’er Yanbian Keli, Wufu Huadu Pian, Fengreqing Koufuye, Zhikechuan Keli, and Neixiao Luoli Pian. It indicates that the functions and main treatments of PG have been supplemented and enhanced in modern times.

## Pharmacology of PLD

### Anti-tumor activity

**FIGURE 2:**
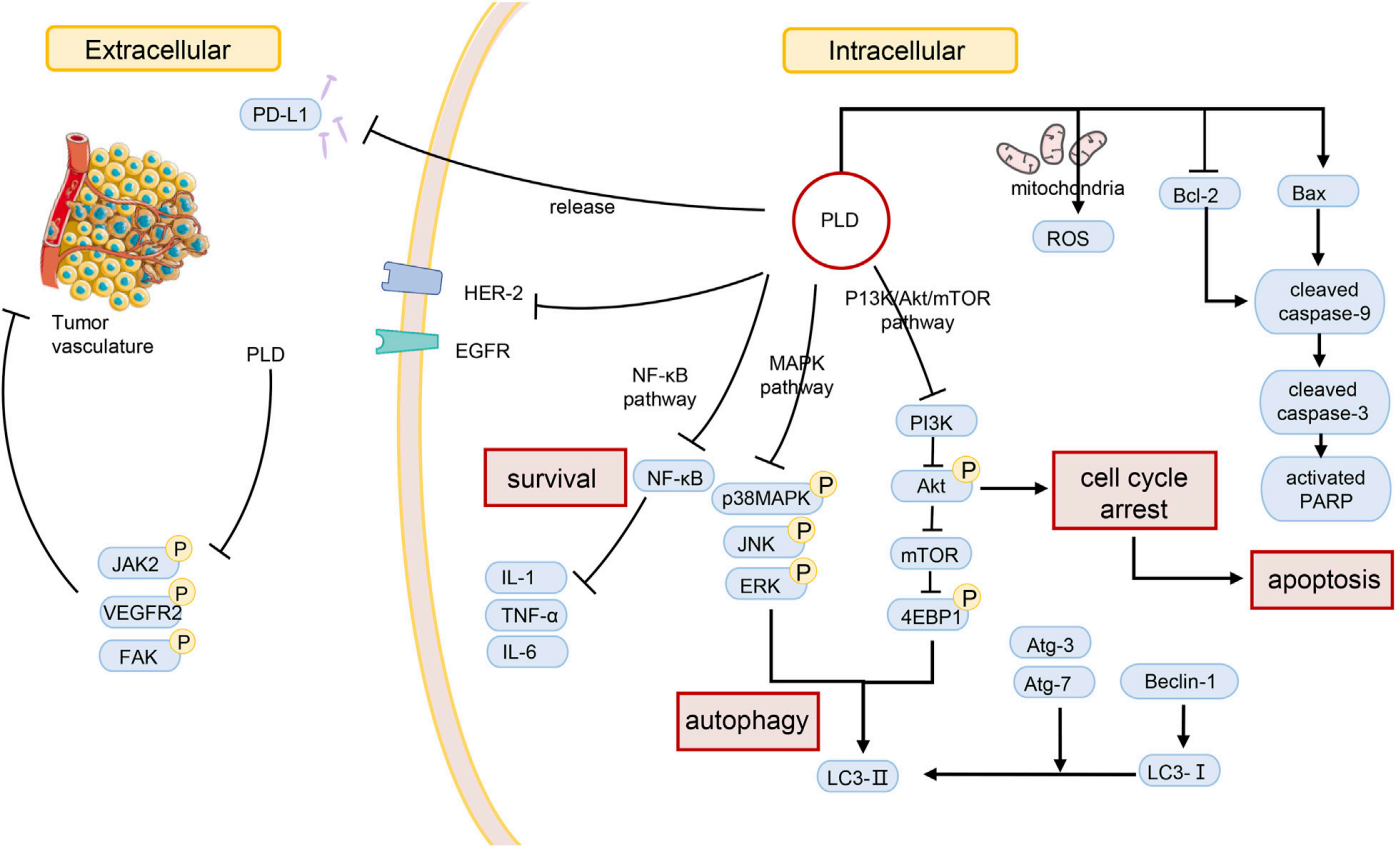
Possible mechanisms of PLD in anti-tumor action.

The prevalence and mortality of cancer are increasing. Coupled with the adverse reactions of chemotherapy, the anti-tumor activity of herbal medicines has been noticed. Extracts of many plants, like PLD, have been identified to have cancer-inhibiting effects. The effectiveness of some herbs and their extracts has been confirmed by *in vivo* and *in vitro* experiments. In the case of PLD, for example, according to pharmacological studies, PLD inhibits tumors in several ways (Table 2). Moreover, the effect concerns multiple processes (Figure 2). PLD induces the antiangiogenic effect outside the tumor cells and decreases the number of microvessels in tumor tissue. Meanwhile, PLD also triggers apoptosis and autophagy in the tumor cells. Multiple signaling pathways were linked to the anti-tumor effect, such as PI3K/Akt/mTOR, MAPK, and NF-κΒ etc. And the mechanism of anti-tumor activity of PLD has been investigated.

**TABLE 2:** The research on the anti-tumor effects of PLD.

Type of tumor	Effects	Mechanism	References
Hepatocellular carcinoma	Inhibiting the proliferation of BEL-7402 cells but also suppresses BEL-7402 xenograft tumor growth	Inducing apoptosis and triggers ERK- and JNK-mediated autophagy in human hepatocellular carcinoma BEL-7402 cells	Li et al. (2015a)
	Triggering a protective autophagy in HepG2 cells	Triggering autophagy through activation of extracellular signal-regulated kinase in hepatocellular carcinoma HepG2 cells	Li et al. (2015b)
	Inducing G2/M arrest and apoptosis in human hepatoma HepG2 cells	Modulating the PI3K/Akt pathway	Qin et al. (2014)
	Reversing histone deacetylase inhibitor resistance in hepatocellular carcinoma cells	Repressing ERK1/2-mediated cofilin-1 phosphorylation	Hsu et al. (2021)
	Inducing autophagy in NCI-H460 and A549 cells	Inhibiting PI3K/Akt/mTOR signaling pathway and activating JNK and p38 MAPK signaling pathways	Zhao et al. (2015)
Lung cancer	As a novel Hsp90 inhibitor	Disrupting Hsp90/Cdc37 complex and enhancing the anticancer effect of mTOR inhibitor	Li et al. (2017)
	Potentiating proliferation inhibition and apoptosis induction upon AKT inhibition	Via feedback blockade in non-small cell lung cancer cells	Li et al. (2016)
	Changing the microenvironment of tumor immunosuppression	Triggering the extracellular release of programed death Ligand-1 in lung cancer cells	Huang et al. (2019)
Breast cancer	Combination treatment with platycodin D and osthole inhibits cell proliferation and invasion in mammary carcinoma cell lines	Mediated by perturbations in the TGF-β/Smads pathway	Ye et al. (2013)
	Inhibiting migration, invasion, and growth of MDA-MB-231 human breast cancer cells	Suppression of EGFR-mediated Akt and MAPK pathways	Chun and Kim (2013)
	Inhibiting S100A8/A9-induced inflammatory response in 4T1 cells	Suppressing the expression of IL-6, IL-1β, and TNF-α *via* inhibition of NF-κB signaling pathways	Ye et al. (2019)
	Enhancing the anti-proliferative effects of doxorubicin on breast cancer MCF-7 and MDA-MB-231 cells	----	Tang et al. (2014a)
	Blocking breast cancer-induced bone loss	Suppressing the formation, activity, and survival of osteoclasts, as well as the growth of metastatic breast cancer cells	Lee et al. (2015)
Gastric cancer	Inducing anoikis and caspase-mediated apoptosis	Via p38 MAPK in AGS human gastric cancer cells	Chun et al. (2013)
Prostate cancer	Decreasing the dosage and side effects of DTX and optimize its clinical use for prostate cancer	Synergistically suppressing cell growth in DU-145 by enhancing apoptosis and alleviating autophagy	Jin et al. (2021)
	An Akt inhibitor or FOXO3a agonist	PD and sorafenib may exert potent anti-cancer effects specifically *via* FOXO3a	Lu et al. (2021)
Glioma	Inhibiting the proliferation, induce the apoptosis and cause the cell cycle arrest in human glioma U251 cells	May be related to the inhibition of PD on the activation of PI3K/Akt signaling pathway	Xu et al. (2014a)
	Inhibiting autophagy and increaseing glioblastoma cell death	LDLR upregulation	Lee et al. (2021)
Pheochromocytoma	Inducing apoptosis and autophagy in PC-12 cells through	Mitochondrial dysfunction pathway	Zeng et al. (2016)
Intestinal cancer	Inhibiting tumor growth by antiangiogenic activity	Blocking VEGFR2-mediated signaling pathway	Luan et al. (2014)
	Sensitizing KRAS-mutant colorectal cancer cells to cetuximab	Inhibiting the PI3K/Akt signaling pathway	Liu et al. (2022a)
Bladder cancer	Inducing ROS-mediated inactivation of the PI3K/Akt/mTOR signaling	Blocking the growth of bladder urothelial carcinoma cells	Park et al. (2022)

### Hepatocellular carcinoma

First of all, PLD has been identified to suppress cancer cell proliferation in multiple studies. PLD significantly reduced cell proliferation of BEL-7402 with IC50 values of 37.70 ± 3.99 mol/L at 24 h (Li et al., 2015a). As a result, PLD treatment led to apoptosis and autophagy. Increasing the Bax/Bcl-2 ratio and the levels of cleaved PARP and cleaved caspase-3, the effect of inducing apoptosis was proved to be dose-dependent (Li et al., 2015a). Additionally, increased amounts of LC3-II and MDC-positive cells were also observed. According to research, the ERK and JNK pathways were critical in the induction of autophagy by PLD. The relative tumor volume was lessened in BEL-7402-bearing mice with PLD treatment (Li et al., 2015a). Furthermore, PLD also was proved to function in human hepatocellular carcinoma HepG2 and Hep3B cells. It was observed that increasing the number of MDC-positive cells and the level of LC3-II expression, and the existence of autophagosomes with a double membrane. The results supposed that PLD could significantly trigger autophagy in multiple cells lines (Li et al., 2015b). Qin et al. (2014) observed that PLD mediated cell cycle G2/M arrest *via* inducing apoptosis in Hep2 cells. The inactivation of the PI3K/Akt signaling pathway was significant for PLD-triggering apoptosis. In Hsu’s study, the combination of PLD and a kind of HDACi was used to treat drug-resistant HCC cells. And the results of the MTT assay supposed the combination significantly reduced apoptosis *via* the cleavage of caspases 9 and 3, and cytochrome C, and the reduction in the phosphorylation of ERK1/2 and CFL-1. And HDACi resistance was treated by suppression of ERK1/2 (Hsu et al., 2021). It seems that PLD could be benefit in inhibiting the resistance to anti-cancer drugs. Efficient endosomal escape after cellular uptake is a major challenge for the clinical application of therapeutic proteins. To overcome this obstacle, several strategies have been used to help protein drugs escape from endosomes without affecting the integrity of the cell membrane. Lei et al. (2022) found that PD were used to greatly enhance the anti-tumor therapeutic effect of protein toxins. In addition to its endosomal escape effect, PD also synergizes with ribosomal inactivation protein to induce apoptosis in hepatoma cells through AKT and MAPK signaling pathways.

### Lung cancer

Autophagy and apoptosis have been described to serve a virtual role in cancer suppression. Zhao et al. (2015) confirmed that PLD triggered autophagy in NSCLC cells through inhibiting the PI3K/Akt/mTOR signaling pathway and activating the JNK and p38 MAPK signaling pathways. And the signaling pathways were investigated in subsequent studies. After co-culture of MK2206 (AKT inhibitor) and PLD for 48 h, cell proliferation of A549 and NCI-H1975 was quelled, and the proportion of apoptotic cells increased. This combination therapy facilitated apoptosis in tumor cells, and the mechanism involved suppression of 4E-BP1 phosphorylation (Li et al., 2016). In a further study, the researchers revealed that PLD could suppress the activity of Hsp90 without inducing Hsp70 (Li et al., 2017). Overcoming the feedback activation of the RTKs/AKT pathway, the combination of an mTOR inhibitor and PLD could result in the blockage of the downstream survival signal in NSCLC (Li et al., 2017). Consequently, PLD is considered a novel Hsp90 inhibitor with anti-cancer effects (Li et al., 2017). Moreover, PD treatment significantly reduced the cell viability, decreased the number of colonies, impaired the mitochondrial function, and induced apoptosis in non-small cell lung cancer (NSCLC) cells. PD can upregulate PUMA (p53 upregulated modulator of apoptosis) to induce apoptosis through JNK1/AP-1 axis in NSCLC (Chen et al., 2022). Moreover, PD could inhibit A549 cell proliferation and induce cell apoptosis by regulating p53/VEGF/MMP2 pathway, in which RRM1 plays an important role directly (Li et al., 2022). On the other hand, PLD can alter the immunosuppressive environment to dampen tumor survival. Programmed death-ligand 1 (PD-L1) expressed on the surface of tumor cells triggers immunosuppression, which restricts the damage to tumor cells. Huang and his co-workers noticed that the protein level of PD-L1 decreased after PLD treatment of lung cancer cells, but the mRNA level of PD-L1 did not decrease (Huang et al., 2019). As a result, the researchers measured the protein of PD-L1 in the cell culture medium. As they predicted, an increase in PD-L1 protein was detected in the cell culture medium. It was confirmed that PLD triggered the extracellular release of PD-L1 (Huang et al., 2019). The researchers suspected that PLD contributed to the release of PD-L1 due to the membrane perturbation effect of PLD (Bailly and Vergoten, 2020). And this effect should be correlated with cholesterol (Bailly and Vergoten, 2020). Zheng et al. (2022) also identified the PD-mediated RNA regulatory network in NSCLC through the whole transcriptome analysis. That is to say, PD inhibits cell proliferation, arrests the cell cycle, and induces cell apoptosis through targeting BCL2-related proteins.

### Breast cancer

Tang et al. (2014b) reported that PLD increased the intracellular concentration of DOX in MDA-MB-231 cells with estrogen receptor-negative. The combination treatment group (DOX and PLD) had higher intracellular DOX concentrations compared to the treatment alone. It implies that PLD increased the uptake of DOX by the cells in Tang’s study. And in another study, PLD combined with Austin enhanced proliferation inhibition and invasion blockade compared with PLD alone. Furthermore, the combination significantly lowered the expression of TβRII, Smad2, Smad3, and Smad4 and blocked TGF-β-induced phosphorylation of Smad2 and Smad3, demonstrating that the TGF-β/Smads pathway was disturbed (Ye et al., 2013). Altogether, PLD in combination with other drugs can show significant effects on breast cancer, which mechanisms are related to the increasing of intake, the inhibition of proliferation, and the blocking of invasion. PLD has been proven to have a blocking effect on invasion and migration. In Chun’s study (Chun and Kim, 2013), the inhibitory effect of migration and invasion was examined by wound healing and a transport chamber assay. After treating MDA-MB-231cells with 15 μM PLD for 24 h, cell migration was quelled by 75%, and the number of cells invading the lower chamber was quelled by 74% (Chun and Kim, 2013). In a further study, the number of cells attached to fibronectin, collagen type I, laminin, and fibrinogen was observed to be significantly reduced (Chun and Kim, 2013). Additionally, they revealed that PLD inhibited cell invasion by decreasing MMP-9 enzyme activity and mRNA expression (Chun and Kim, 2013). Based on later findings, this was not the only way to inhibit invasion and migration. The nuclear translocation of NF-κB p65 was diminished under the intervention of PLD. And the expression of TNF-α, IL-6, and IL-1β was also suppressed in 4T1 cells. PLD represses the invasion and migration of 4T1 cells by inhibiting inflammation (Ye et al., 2019). PLD has been investigated to exert a significant part in restraining breast cancer-induced bone destruction. And bone is one of the targets of advanced distant metastasis in breast cancer (Jin et al., 2018; Pedrosa et al., 2018; Shen et al., 2021). When bone marrow macrophages (BMMs), the osteoclast precursors, were exposed to PLD for 5 days, cell viability decreased by 21% and 86% at 5 and 10 µM (Lee et al., 2015). In the presence of 1 µM PLD, osteoclast production was significantly suppressed by blocking NF-κΒ, ERK, and p38 MAPK activation (Choi et al., 2017). Accordingly, PLD is recognized by researchers as an anti-osteoporotic candidate for the management of osteoporotic diseases (Choi et al., 2017). VEGF and IL-8 are potent angiogenic factors and contribute to tumor angiogenesis by directly and indirectly promoting angiogenic processes, including the proliferation, adhesion, migration, and tube formation of endothelial cells. Son et al. (2022) found that PD can inhibit VEGF-induced angiogenesis by blocking the activation of mitogen-activated protein kinases and the production of IL-8. Excessive neutrophils facilitate breast cancer growth and pulmonary metastasis. Increased PD-L1 inhibits spontaneous apoptosis of neutrophils. Ye et al. (2023) found that PD inhibits the expression and positive rate of PD-L1 in neutrophils to induce neutrophil apoptosis and decrease their high migratory capacity. In conclusion, the administration of PD inhibited the PI3K/Akt signaling pathway by reducing the expression of PD-L1 in neutrophils. PD promoted neutrophil apoptosis, thereby inhibiting the establishment of a premetastatic niche and ultimately blocking the development of pulmonary metastasis.

### Prostate cancer

Researchers observed that PLD boosted apoptosis and cell cycle arrest induced by sorafenib in PC3 cells (Lu et al., 2021). The protein and mRNA expression of FOXO3a, a downstream target of Akt, was increased due to PLD therapy. A synergistic effect of PLD and sorafenib was found to be evident, although affecting the downstream target of the PI3K/Akt axis (Lu et al., 2021). Meanwhile, treatment with PLD resulted in the activation of FOXO3a and increased expression of Fasl, Bim, and TRAIL expressions (Lu et al., 2021). Additionally, in Jin’s study, PLD could also accelerate apoptosis induced by other drugs like docetaxel. It was observed that cleaved PARP, caspase-3/9, and Bax/Bcl-2ratio were upregulated in the combination treatment group (Jin et al., 2021).

### Glioblastoma multiforme

Xu et al. (2014b) studied on the effects of PLD on human glioma U251 cells. According to their results, the PI3K/Akt signaling pathway was activated in the early and late apoptotic rate, which caused the increase of apoptotic index and the level of pro-apoptotic proteins. And the increase of the proliferation inhibition rate was exerted in a dose- and time-dependent manner. Lee et al. (2022) discovered that PLD could be a potent anti-glioblastoma multiforme (GMB) drug. However, an opposing viewpoint was proposed that PLD was an autophagy inhibitor. Researchers found that the LC3B-II level was increased while the p62 level was increased simultaneously. The autophagy regulation was related to a high abundance of LDLR in GBM cells instead of *via* the PI3K/AKT/mTOR or MAPK signaling pathway. It was PLD that promoted the uptake and accumulation of cholesterol in lysosomes so that the death of GBM cells was led (Lee et al., 2021).

### Intestinal cancer

Chun et al. (2013) explored the effects of PLD on various cancer cell lines. According to the results, the IC_50_ value of PLD against Caco-2 cells was 24.6 μM. And the ratio of cells in the sub-G1 phase was increased. Luan et al. (2014) verified that PLD could contribute to cancer inhibition by inhibiting angiogenesis in a dose-dependent way. PLD inhibited HUVEC tube formation at a concentration of above 0.3 μM and inhibited the motility of HUVECs at a concentration of higher than 10 μM. Moreover, PLD exactly decreased microvessel density and delayed the growth of HCT-15 xenograft. Moreover, Platycodin-D can regulate Akt in different trends based on tissue types. Liu et al. (2022a) provided a potentially reliable theory for the improvement of Cetuximab chemotherapy efficacy with PD treatment. Specifically, PD may sensitize KRAS-mutant colorectal cancer cells to cetuximab *via* inhibition of the PI3K/Akt signaling pathway.

### Other tumors

PLD had the cell cycle arrest effect on PC-12 cells (Zeng et al., 2016) and PC3 cells (Lu et al., 2021) at the G0/G1 phase. The mechanism of the anti-tumor effect was proved to involve multiple routes. In Zeng’s study, PLD showed significant anti-cancer effects on PC-12 cells with the IC_50_ value of 13.5 ± 1.2 μM at 48 h (Zeng et al., 2016). PLD increased the levels of ROS and induced a decrease in mitochondrial membrane potential. And then, mitochondrial dysfunction occurred in PC-12 cells, leading to cell apoptosis (Zeng et al., 2016). PD also had chemopreventive potential through the induction of ROS-dependent apoptosis in PC3 cells, and that this compound could be useful for developing an effective and selective natural source to inhibit cancer cell proliferation (Choi, 2022). Furthermore, in Zeng’s study, the IC_50_ value of PLD against SGC-7901 was 18.6 ± 3.9 μM (Zeng et al., 2016). Chun et al. (2013) discovered that AGS cells were significantly sensitive to PLD. And PLD treatment induced AGS cells rounding and detaching from the plate, causing anoikis-like apoptotic cell death. In a further study, the mechanism was related to the extrinsic pathway *via* Fas-L and the intrinsic pathway *via* mitochondrial Bcl-2 family members. Meanwhile, the activation of p38 MAPK played a crucial role in cell apoptosis induced by PLD. In addition, PD blocked the growth of bladder urothelial carcinoma cells by inducing ROS-mediated inactivation of the PI3K/Akt/mTOR signaling (Park et al., 2022).

### Anti-inflammatory activity

Inflammation is a physiological process necessary for homeostasis. And upregulation of cytokine/chemokine release is one of its hallmarks (Kubatka et al., 2021). The NF-κB signaling pathway is crucial to the development of inflammatory processes. The NF-κB signaling pathway is activated and the expression of genes that the inflammatory response is initiated. Subsequently, the release of pro-inflammatory cytokines, chemokines, and additional inflammatory mediators from innate immune cells is induced (Tabas and Glass, 2013; Liu et al., 2017). Then organ fibrosis (Wynn and Ramalingam, 2012) and inflammasome activation (Liu et al., 2017) are encouraged. On the other hand, chronic inflammation is seen as a sign of cancer initiation and progression. Immune cells release cytokines and growth factors in the tumor microenvironment (TME) to increase the proliferation of cancer cells and resistance to cell death and stresses. Pre-malignant and cancer cells could be directly affected to promote tumor growth and progression (Greten and Grivennikov, 2019). The effect of PLD to inhibit cytokine/chemokine release has been demonstrated. In addition to the NF-κB signaling pathway, PLD also affects other signaling pathways to reduce inflammation.

### The disease of the respiratory system

**FIGURE 3:**
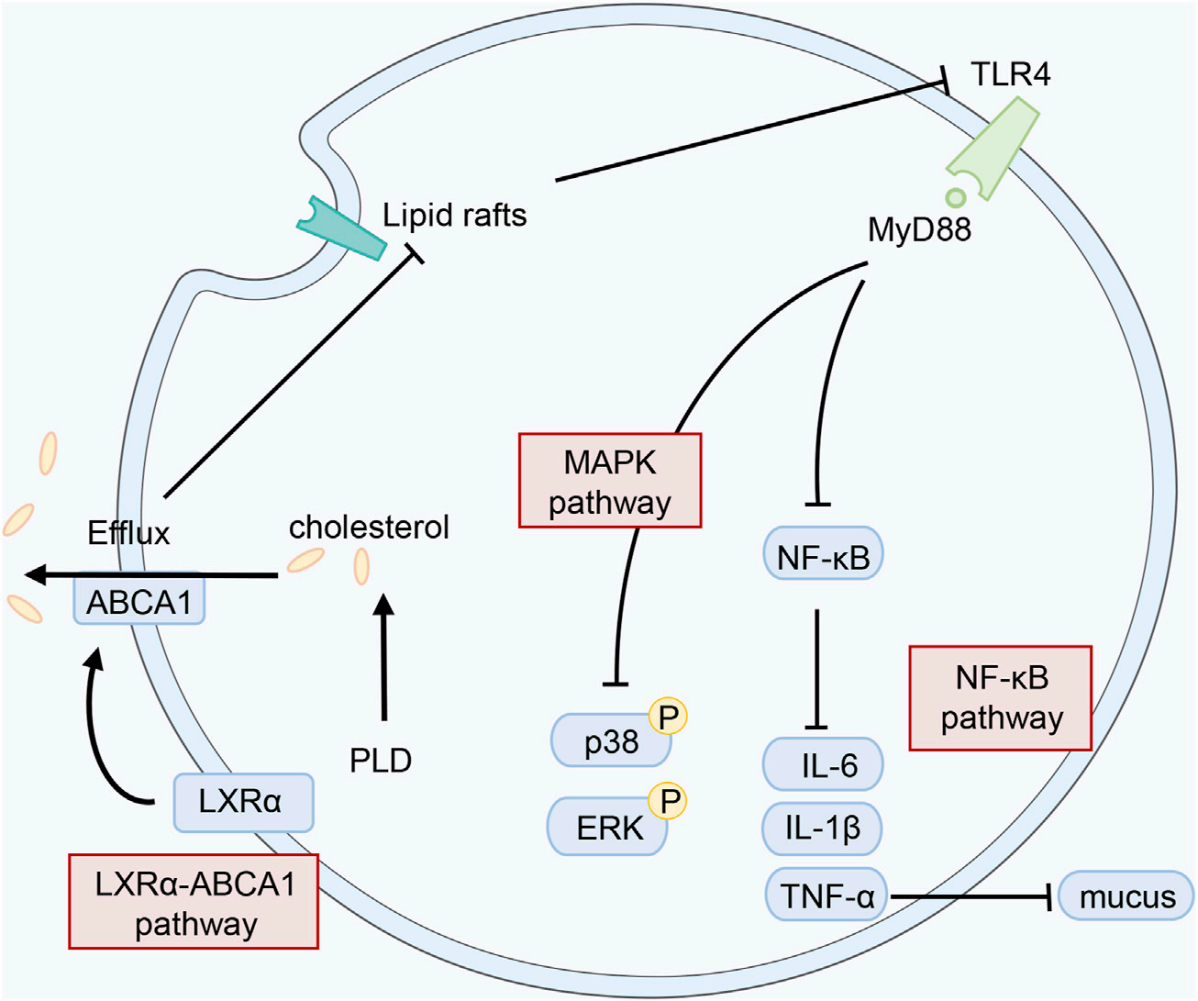
Possible mechanisms of PLD in anti-inflammatory action.

PLD markedly quells exogenous and endogenous inflammation, particularly in the respiratory tract. Studies have demonstrated that PLD reduces the levels of pro-inflammatory cytokines (IL-1, IL-6, TNF-α, etc.) through multiple pathways, such as NF-кBp65 (Zhang et al., 2015b; Wang et al., 2016) and IGF-1R/PI3K/Akt (Suh et al., 2018). According to the references, PLD exerts an essential function in treating respiratory inflammation and reducing mucus secretion in the respiratory tract through various signaling pathways. These processes are shown in Figure 3.

In treating acute lung injury, PLD lessens the protein levels of NF-κΒ, Caspase-3, and Bax to decrease inflammatory cell infiltration and pathological inflammation (Tao et al., 2015). Additionally, PLD exhibited a protective effect against cigarette smoke-induced lung inflammation by quelling inflammatory and oxidative responses. And the Nrf2 signaling pathway was activated to reduce inflammatory cells infiltration as well as TNF-α and IL-1β production in this process (Gao et al., 2017). In cough reflex guinea pigs induced by citric acid treatment, fermented PG extracts (FPE) with an elevated PLD content significantly reduced the number of coughs (Lee et al., 2020). PLD also disrupts lipid rafts and blocks TLR4 translocation into lipid rafts by activating the LXRα–ABCA1 signaling pathway (a significant pathway for reducing inflammation) (Fu et al., 2017a). Hu et al. (2016) discovered that PLD disrupted the formation of lipid rafts by depleting cholesterol. The effect of attenuating lung histopathologic changes, myeloperoxidase activity, and pro-inflammatory cytokines levels was achieved. Increasing mucus secretion is one of the symptoms of chronic bronchitis. And it is caused by the damage of the epithelium of the central airways, infiltration of inflammatory cells, and hypertrophy of smooth muscle cells (Deng et al., 2020). In a later study, PLD suppressed the expression levels of TLR4, MyD88, p-IκBα, p-p65, and the NLRP3 inflammasome, reducing mucus secretion (Wu et al., 2021). Lee et al. (2010a) revealed that PLD quelled EGF, PMA, and TNF-α-induced MUC5AC gene expression at concentrations of 10^−4^ M, resulting in reduced production of MUC5AC mucin. In other words, PLD could suppress mucus secretion in the respiratory tract, which supports the traditional use of PG. Furthermore, PLD demonstrated powerful anti-pneumonia effects attributed to *Mycoplasma pneumoniae.* Following the study, PLD exerted a sharp function in boosting cell growth after anti-*M. pneumoniae* treatment (Meng et al., 2014). PLD has considerable ability to quell *M. pneumoniae* with a MIC of 16 μg/ml (Meng et al., 2014). It was demonstrated that PLD suppressed the adhesion of *M. pneumoniae* to epithelial cells *via* lowering the expression of P1 and P30 mRNA levels (Meng et al., 2017).

### Liver injury

There are numerous studies on the impact on the liver. PD significantly lowered the levels of pro-inflammatory cytokines attributed to alcohol exposure (Li et al., 2015c) and diabetes mellitus (Chen et al., 2015), including tumor necrosis TNF-α, interleukin -1β, and IL-6. Treatment with PLD resulted in a significant decrease in serum levels of triglyceride (TG), total cholesterol (TC), and low-density lipoprotein cholesterol (L-DLC) (Li et al., 2015c). Kim et al. (2013a) identified that PLD alleviated liver injury and fibrosis in mice by weakening oxidative stress. Additionally, serum alanine aminotransferase, serum aspartate aminotransferase, and total bilirubin levels were lower in the PLD-treated group compared to bile duct ligation (BDL) alone. Li et al. (2015c) reported that PLD lowered the liver coefficient and the levels of TG, TC, and L-DLC at doses of 10 and 20 mg kg^−1^. Furthermore, in their experiments, PLD exhibited a significant effect in lowering the levels of inflammatory factors and modifying the activity of antioxidant enzymes. Similar results could be observed in other studies. Chen and his co-workers demonstrated that PLD (10, 50, and 100 mg kg^−1^) modulates the balance of Th17 and Treg cells in liver tissues. And Treg cells were increased in the liver and spleen (Chen et al., 2015). PLD also quelled the phosphorylation of JAK and STAT-3 and the expression of RORγt and increased the expression of Foxp3 (Chen et al., 2015). What’s more, PLD affected the expression level of apoptotic proteins and autophagy-relevant proteins. It was also proved that PLD alleviated liver fibrosis and activation of hepatic stellate *via* the JNK/c-Jun pathway (Liu et al., 2020). And the phosphorylation of JNK and c-Jun played an essential role in the underlying molecular mechanism. Wen et al. (2022) also found that PD ameliorated NAFLD *in vitro* by reducing oxidative stress and stimulating autophagy.

### Other inflammatory diseases

The anti-inflammatory activity of PLD is demonstrated not only in respiratory diseases but also in other organs. Like skin inflammation, PLD could inhibit the NF-кB signaling pathways to address atopic dermatitis (Choi et al., 2014) and acne (Suh et al., 2018). Besides, the PI3K/AKT and AMPK signaling pathways were also influenced by PLD in the treatment of colitis (Guo et al., 2021). In the study on arthritis, the levels of TNF-α and IL-6 were decreased (Kwon et al., 2014), and the expression of LXRα was increased in the PLD-treated group (Qu et al., 2016). The suppression of NF-κΒ and activation of LXRα allowed PLD to repress mastitis as well (Wang et al., 2017). Besides, Fu et al. (2017b) demonstrated that PLD could silence inflammation in rat microglia by activating the LXRα-ABCA1 signaling pathway to induce cholesterol flow. Zhang et al. (2021a) found that PLD decreased the production of TNF-α, IL-1β, and IL-6 in BV-2 cells. Additionally, Kim et al. (2018) discovered that platycodin could heal muscle injury by lowering the levels of serum lactate dehydrogenase (LDH), creatinine kinase (CK), and C-related protein and suppressing matrix metalloproteinase (MMP). The PI3K/mTOR signaling pathway was found to be inactivated, thereby restraining the activation of the full immune cell by inhibition of the pro-inflammatory cytokines, as revealed by the results indicating the prevention of the high-glucose-induced inflammation response by PD (Liu et al., 2022b). PD3 treatment alleviated the airway remodeling and inflammation in asthmatic mice, which might be related to downregulating the phosphorylated proteins in the MAPK/NF-kappa B signaling pathway (Peng et al., 2022).

### Anti-viral activity

Numerous studies have proven that herbal remedies are worthwhile to study, and plants and their extracts have excellent anti-viral properties. The author observed that the majority of the studies on the anti-viral effects of PLD focused on RNA viruses. Taking SARS-CoV-2 as an example, the prevention and treatment of COVID-19 is a global challenge. In order to support viral replication, the virus infects normal cells through internalization and impacts metabolic pathways in cells, including alterations in fatty acid metabolism, mitochondrial respiration, and pyrimidine nucleotide biosynthesis. And the cell cycle, nucleic acid metabolism, and immune signaling are also disrupted (Hekman et al., 2020). Li and his colleagues reported that SARS-CoV-2 infection of lung epithelial cells induced caspase-8 activation, which caused cell apoptosis and inflammatory cytokines activation (Li et al., 2020). Additionally, inflammatory responses were mediated through the SARS-CoV-2-induced necroptosis pathway (Li et al., 2020). Other pathways are also impacted, such as tight junction organization and lipid metabolism (Hekman et al., 2020). Although PLD showed intense activity against viruses, further research is required to verify these findings, and clinical research is lacking.

### SARS-CoV-2

SARS-Cov-2 was discovered in late December 2019 (Lu et al., 2020). Besides, it has 89.1% nucleotide similarity to SARS (Wu et al., 2020). Patients will have a fever, cough, and chest discomfort (Zhu et al., 2020). Statistically, patients over 65 with comorbidities and acute respiratory distress syndrome (ARDS) have an increased risk of death (Yang et al., 2020b). The virus has mutated several times to make matters worse due to the accumulation of high numbers of mutations, especially in the spike protein (Khandia et al., 2022). These variants are known as Alpha, Beta, Gamma, Delta, Omicron, etc. Kim et al. (2021) reported two pathways of PLD effectively blocked SARS-CoV-2 infection, including lysosome and transmembrane protease serine 2 (TMPRSS2)-driven entry. PLD functions by redistributing membrane cholesterol to prevent membrane fusion. An hour PD treatment effectively decreased pSARS-CoV-2 entry in a dose-dependent manner, with a half-maximal inhibitory concentration (IC50) of 0.69 μM in ACE2+ cells. PLD showed a strong ability against SARS-CoV-2 infection in *in vitro* experiments. In order to further understand the structural activity relationship between saponin-based antiviral drugs against SARS-CoV-2, Jang et al. (2022) discovered that the C3-glucose, the C28-oligosaccharide moiety that consist of (-> 3)-beta-D-Xyl-(1 -> 4)-alpha-L-Rham-(1 -> 2)-beta-D-Ara-(1 & RARR) as the last three sugar units, and the C16 -hydroxyl group were critical components of saponin-based coronavirus cell entry inhibitors. These findings enabled us to develop minimal saponin-based antiviral agents that are equipotent to the originally discovered PD. The newly developed compounds inhibit the SARS-CoV-2 entry by blocking the fusion between the viral and host cell membranes. The effectiveness of the newly developed antiviral agents over various SARS-CoV-2 variants hints at the broad-spectrum antiviral efficacy of saponin-based therapeutics against future coronavirus variants. RdRp is an important therapeutic target of SARS-CoV-2. PD has a strong inhibiting effect against SARS-CoV-2 with IC50 values of 619.5. And its inhibitory effect is highly likely through competitive prevention of the RNA template-primer RNA entering into its RdRp binding cavity (Zhang et al., 2022).

### Type 2 porcine reproductive and respiratory syndrome virus (PRRSV)

Zhang et al. (2018) found that the 50% effective concentrations (EC50) of PLD against the four PRRSV strain infections ranged from 0.74 to 1.76 µM. The potent inhibition against PRRSV infections was related to attachment and internalization blocking, RNA replication and release inhibiting, and the cell-to-cell transmission pathways blocking. Moreover, PLD (2, 4, and 8 µM) significantly weakened the ability of PRRSV to infect MARC-145 cells in a dose-dependent manner by co-incubated with the virus. Zhang et al. (2018) counted that PLD did directly interact with PRRSV particles.

### Hepatitis C virus (HCV)

A modeling study (Blach et al., 2017) reported that there were 711 million patients worldwide. Furthermore, hepatocellular carcinoma (HCC) is a possibly severe complication in the natural history of chronic hepatitis C (Conti et al., 2016). However, the resistance to direct-acting anti-viral (DAA) drugs is a main reasons for interferon-free treatment failure (Pawlotsky, 2016). Although DAA could induce the resolution of HCV infection, the occurrence of HCC could not be reduced by using DAA (Conti et al., 2016). Kim et al. (2013b) researched the inhibition ability of triterpenoid saponins in PG on HCV RNA replication. Moreover, the inhibition of PLD occurred in a dose-dependent manner with an IC50 value of 5 μg/ml.

### Anti-oxidation activity

The balance of antioxidants and oxidants plays a significant part in keeping the body working well. Oxidative stress can cause damage to cells, and the mechanism is complex. Oxidative damage is present in various diseases, such as atherosclerosis (Zhang et al., 2021b; Mushenkova et al., 2021), and neurodegenerative diseases (Collin, 2019). While in Ryu’s study, the antioxidant activities of various saponins isolated from PG were compared. PLD exhibited the most significant total oxidant scavenging capacity (TOSC) values for peroxyl radicals, followed by polygalacic acid, platycodigenin, deapio-platycosides E, and platycoside E. And the antioxidant activity of agarose differs due to its structure and the number of attached sugar residues (Ryu et al., 2012). It has been demonstrated that PLD significantly suppressed the elevation levels of superoxide dismutase (ROS) and malondialdehyde (MDA) (Zhang et al., 2021a). Shi et al. (2020) revealed that PLD increased the potential membrane ratio and stimulated the proliferation of mitochondrial mass. Nuclear respiratory factor and mitochondrial transcription factor A were increased in mitochondrial biogenesis. In their study, PLD significantly decreased the levels of ROS, 4-hydroxynonenal (4-HNE), and MDA in H_2_O_2_-treated 2BS cells and reversed senescence-like phenotypes.

Increasing antioxidant enzymes, such as glutathione (GSH), glutathione peroxidase (GPx), and superoxide dismutase (SOD), is a vital way to protect nephridium from cisplatin-induced nephrotoxicity. It is in this way that PLD exerts its nephroprotective effects. Pretreatment with PLD decreased nitric oxide (NO), lipid peroxidation, and increased antioxidant enzymes in mice. And the elevation in serum blood urea nitrogen (BUN) and creatinine (CRE) levels by 85% and 83%. It has been suggested that PLD significantly protects the kidney (Kim et al., 2012). Hu et al. (2021) revealed that PLD (0.25, 0.5, and 1 μM) could dose-dependently alleviate oxidative stress. Furthermore, PLD treatment reversed the elevation of apoptosis induced by cisplatin (Hu et al., 2021). It was PLD pretreatment that reversed the increase of the production of ROS and malondialdehyde and the decrease in the activities of SOD and catalase (CAT) induced by H/R (Wang et al., 2018). Additionally, a similar phenomenon was shown in another study in which PLD (10, 20, 40 μM) attenuated oxidative stress with increasing doses (Wang et al., 2019b).

### Hypoglycemic effect

Abnormally high blood glucose levels are the main characteristic of T2D. Modern scholarly research has found that reasonable control of blood glucose could reduce glucose-induced toxicity and improve islet cell function, preventing and delaying the progression of diabetic complications. PD treatment might regulate the hepatic gluconeogenesis pathway with the increased phosphorylation/expression of AMPK and decreased expressions of PCK1 and G6Pase. In the aspect of lipid metabolism, PD decreased the whole-body lipid levels, including total cholesterol (TC), triglycerides (TG), and high-density lipoprotein (HDL), and reduced the hepatic fat accumulation induced by T2D through the AMPK/ACC/CPT-1 fatty acid anabolism pathway. In addition, PD may have a potential direct effect on AMPK and other key glycolipid metabolism proteins. To summarize, PD modulation of hepatic glycolipid metabolism abnormalities is promising for T2D therapy in the future (Shen et al., 2023). Diabetes mellitus is a group of physiological dysfunctions associated with hyperglycemia-mediated oxidative stress and apoptosis in pancreatic β-cells. PD can protect INS-1 cells from STZ-induced oxidative stress and apoptosis. The protective effect of PLD might be ascribed to the regulation of p38 and Nrf2 pathways (QIAO et al., 2021). Moreover, PD suppressed ferroptosis in high glucose-induced cells by regulating GPX4 expression, suggesting that PD may help treat diabetic nephropathy. However, more studies are needed to apply PD to clinical treatment of diabetic nephropathy (Huang et al., 2022). High glucose (HG) induces excessive ROS production in oral mucosal cells, and induces apoptosis to cause cell damage. High glucose also induces excessive ROS production in oral mucosal cells, and induces apoptosis to cause cell damage. Through the downregulation of PI3K and mTOR, PD was found to be able to mitigate the inflammatory responses of the HG-induced oral mucosal cells, as demonstrated by the results. Albeit further studies would still be needed to investigate the exact role of PD in oral inflammatory responses, these findings could, as of now, provide a novel strategy in the treatment of oral diseases (Liu et al., 2022b).

### Anti-obesity activity

According to an analysis of the trends of overweight and obesity between 1980 and 2015, more than two-thirds of deaths related to high body-mass index (BMI) were due to cardiovascular disease (Collaborators et al., 2017). Moreover, in a study on COVID-19 patients, obesity was one of the factors associated with high mortality in hospitals (Docherty et al., 2020). The anti-obesity effect of PLD has been demonstrated to involve interacting with various signaling factors. Lee et al. (2010b) reported that PLD inhibited intracellular triglyceride accumulation in 3T3-L1 cells with an IC50 of 7.1 μM. And the positive impact was found to involve the upregulation of Kruppel-like factor (KLF)2 and subsequent downregulation of Peroxisome proliferator-activated receptor (PPAR)γ. These effects further contributed to downregulations of lipid metabolizing enzymes and reduced intracellular triglyceride accumulation. Besides, PLD was responsible for the accumulation of β-catenin in the nucleus, and the upregulation of the target genes, cyclin D (CCND) 1 and PPARγ (Lee et al., 2011). They demonstrated that PLD inhibited adipogenesis by stabilizing β-catenin and the resulting downregulation of adipogenic factors. In another study (Lee et al., 2012), the effect of PLD on PPARγ2 was also confirmed. Researchers found that PLD significantly reduced fat accumulation and volume *via* the AMPK pathway. PLD could increase AMPKα, similar to AICAR, and reduce PPARγ2 and C/EBPα expression to enhance lipid metabolism. In Kim’s study (Kim et al., 2015), PG ethanolic extract suppressed the differentiation of 3T3-L1 cells by downregulating cellular induction of the PPARγ, C/EBPα, lipin-1, and adiponectin but increased the expression of SIRT1 and the phosphorylation of AMPKα. The inhibition of lipid accumulation by 5 μM of PLD (the main component of PG extract) was 41.01%. More importantly, PLD could downregulate the E3 ubiquitin ligase, leading to increasing the level of the low-density lipoprotein receptor (LDLR) on the cell surface. According to the results of the study, combined treatment of HepG2 cells with simvastatin and PLD had a synergistic effect on improving LDLR expression and LDL-C uptake. It is advantageous for managing atherosclerotic cardiovascular diseases (Choi et al., 2020).

As mentioned above, PLD effectively regulates factors and receptors at the cellular level. Correspondingly, PLD was effective on animals. *In vivo* experiment, the inclusion of PG extract in the high-fat diet (HFD) markedly attenuated food intake, body weight, epididymal fat weight, adipocyte size, and blood glucose levels by the oral glucose tolerance test in mice. Meanwhile, serum levels of adiponectin, resistin, leptin, fructosamine, and triglycerides were maintained (Ahn et al., 2012). PLD increased the phosphorylation of AMPK and acetyl-CoA carboxylase in HFD-fed rats and HepG2 cells, activating AMPK *via* SIRT1/CaMKKβ (Hwang et al., 2013). In another of Kim’s study (Kim et al., 2019), PLD treatment elevated the expressions of critical regulators of brown adipose tissue (BAT), uncoupled protein 1 (UCP1), and peroxisome proliferator-activated receptor γ coactivator 1α (PCG1α), associated thermogenesis. Furthermore, PLD significantly attenuated weight and white adipose tissue weight in db/db mice.

### Organ protection

PLD not only has protective effects on nephridium, liver, and muscle but on the heart. PLD has a significant effect on improving cardiac functional indices. Moreover, it showed a potential to treat hypertension. In Lin’s experiment (Lin et al., 2017), PLD protected cardiomyoblasts from apoptosis occurring in response to hypertension. PLD reduces pHSF1 and pJNK expression induced by angiotensin II (Ang II). And SIRT1 expression was also upregulated by PLD compared with the Ang II group. PLD suppressed apoptosis, as indicated by reductions in Ang II-induced caspase-3 activity and IGF-IIR translocation to the cell membrane. PLD also could decrease the expression levels of the eccentric hypertrophy marker and concentric hypertrophy markers. And the decreased expression levels of NFATc3, p-GATA4, and BNP were also observed (Lin et al., 2018). Research has also proved that PD is a promising natural medicine for intestinal protection. Cisplatin (CP) is a kind of commonly used chemotherapeutic drug in cancer treatment, but it has a variety of adverse reactions, among which gastrointestinal reaction is one of its main limiting toxicities. The intestine is very susceptible to CP chemotherapy, and the intestinal toxicity caused by CP widely affects gastrointestinal function. Fan et al. (2022) found that PD can restore the intestinal mechanicalbarrier by reducing endoplasmic reticulum stress-mediated apoptosis, which might be related to PERK-eIF2ɑ-ATF4 signaling pathway.

### Anti-fibrosis

Abnormal fibroblast proliferation and excessive extracellular matrix (ECM) deposition led to the formation of hypertrophic scars (HSs). Yu et al. (2022) found that PD inhibited the proliferation and migration of HSFs by inhibiting fibrosis-related molecules and promoting apoptosis *via* a caspase-dependent pathway. The TGF-beta/Smad pathway also mediated the inhibition of HSFs proliferation and HSFs differentiation into myofibroblasts. Therefore, PD is a potential therapeutic agent for HSs and other fibrotic diseases.

### Anti-coagulation

It is a threat in front of COVID-19 that microvascular, venous, and arterial thrombosis could be caused by SARS-CoV-2 (McFadyen et al., 2020). In a meta-analysis on the relationship between SARS-CoV-2 and cerebral venous thrombosis (CVT), the symptoms of CVT developed after respiratory in 90% of patients (Baldini et al., 2021). Zhang et al. (2020) found that SARS-CoV-2 enhanced the platelet activation *via* the MAPK signaling pathway and directly stimulated platelets to facilitate the release of coagulation factors. The anti-coagulation of PLD was made clear by using mice platelets. PLD made mice platelets significantly impaired hemostasis and arterial thrombus formation *in vivo*. PLD significantly affected the internalization of glycoprotein receptors α_IIb_β_3_, GPIBα, and GPVI, which was not prevented by GM6001, cytochalasin D, BAPTA-AM, and wortmannin (Lu et al., 2013; Luo et al., 2018; Ceña-Diez et al., 2019).

### Spermicidal activity

#### Pharmacokinetics

Pharmacokinetics studies on PLD showed a short half-live (t_1/2_) and low bioavailability. And the bioavailability of PLD is significantly influenced by the administration route. According to the experiment results, the t_1/2_ of oral administration is 5.42 ± 1.9 h compared to 2.14 ± 0.18 h for intravenous injection. The peak plasma concentration (C_max_) and the time to reach peak plasma concentration (T_max_) were significantly longer for oral than for intravenous (Kwon et al., 2017). While the bioavailability of PLD can be boosted by using it with other herbs, like *Glycyrrhiza uralensis* Fisch. (Gancao in Chinese). PG is frequently prescribed as an herb pair with Gancao, which is widely used in traditional Chinese medicine. And a prescription including PG and Gancao, known for its healing properties, was recorded in *Shanghan Lun* thousands of years ago. From the perspective of modern research, the reasons for the pairing of PG with Gaocao are thought to involve various pharmacokinetics. Compared with PG, the T_max_, t_1/2_, and area under the plasma concentration curve (AUC) of PLD were increased in the herb pair group (Shan et al., 2015). And the bioavailability of liquidity, isoliquiritin, glycyrrhizin, and glycyrrhetinic acid, was improved with the existence of PG (Mao et al., 2017). The mechanism of the combination of PG and Gancao was interpreted in these studies. In addition, other methods have been tested to improve the bioavailability. For example, Shen et al. (2021) selected soybean phospholipids as the surfactant, and ethanol as the cosurfactant to produce a water-in-oil microemulsion. The microemulsion extended the t_1/2_ from 1.97 ± 0.08 h to 4.46 ± 0.90 h, with a relative bioavailability of 165%. Changing dosage forms could be a valuable measure to extend the t_1/2_.

#### Toxicity

New saponins have been isolated and identified over time. And the hemolytic activity of saponins has simultaneously attracted the attention of researchers. Intravenous administration of saponins may cause hemolysis, which is a safety concern restricting clinical application and product development. The 19 saponins studied by Sarikahya revealed hemolytic activity in human erythrocytes (Sarikahya et al., 2018). It is well known that structure determines properties. Hemolytic activity is correlated with the molecular structure of saponins. Compared to ursane or dammarane types, oleanane-type saponins have stronger hemolytic activity (Vo et al., 2017). The hemolytic activity of PLD, an oleanane-type saponin, has been demonstrated. Sun and his colleagues discovered that not only PLD but also other saponins in PG also have hemolytic activity (Sun et al., 2011). Furthermore, it was evidenced that this activity is related to the following elements (Xie et al., 2008; Sun et al., 2011; Vo et al., 2017): 1) the aglycone, 2) the number and linkage of sugar residues attached to C-3 of aglycone, 3) the numbers of glycosides attached to C-28 of the aglycone. The hemolytic activity will be enhanced if there is a second sugar chain attached to the aglycone or if there is a hydroxyl group at position 16 of the olean backbone (Gilabert-Oriol et al., 2013). Altogether, the hemolytic activity of PLD is correlated with its molecular structure. This issue is expected to be solved to boost the security of using saponins.

## Conclusion

As an herbal medicine with a long and extensive history of application, PG has apparent effects in expelling phlegm and purging the pus. Although the biological activities of demonstrated, more in-depth studies on its herbal basis and mechanism of action are still needed. Modern research has revealed that PLD, the main active ingredient of PG, has various pharmacological effects such as anti-inflammatory, anti-viral, antioxidant, anti-coagulation, anti-cancer, and immune regulation. Additionally, PLD stimulates neuronal growth to boost cognitive competence though phosphorylation of the ERK1/2 signaling pathway *in vitro* (Kim et al., 2017). The authors also considered the relationship between traditional uses and modern studies. The anti-inflammatory and antioxidant activities of PLD contribute to the lung heat-clearing effects of PG. Meanwhile, the anti-viral, anti-lung cancer, and organ-protective effects of PLD may reflect the detoxifying effects of PD. Among them, the anti-respiratory inflammatory effect of PLD coincides with the suppressing cough and expectorant phlegm effects of PG. The anti-pneumonia and lung cancer activities and the protection of lung cells corroborate the efficacy of PG in treating lung carbuncle. Other effects, such as anti-obesity and anti-coagulation activity, have not been demonstrated in traditional applications. In the authors’ opinion, it is relevant to limited conditions and different modes of administration in ancient times. On the contrary, these activities have been observed in modern times owing to the development of medical technology. In other words, these activities refine modern technology for traditional uses, which helps to take full advantage of PLD.

As a traditional Chinese medicine with the homology of medicine and food, PG is featured with low toxicity, few side effects, and great development potential. Nevertheless, traditional extraction methods frequently observed a low yield of PLD. The application of PLD is constrained by poor production and complex extraction methods. Several researchers are sparing no effort to boost the yield of PLD with novel methods. Mechanochemical-assisted extraction was proved to be efficient with extracting PLD from roots of PG. The highest yield of PLD *via* mechanochemical-assisted extraction was 7.16 ± 0.14 mg/g (Wu and Xi, 2020). Apart from mechanochemical-assisted extraction, abundant studies have concentrated on the extraction of PLD through enzyme biocatalysis. Studies demonstrated that PLD precursors could be hydrolyzed and converted to PLD (Ahn et al., 2018; Kil et al., 2019; Shin et al., 2019). Extracellular *Aspergillus usamii* β-D-glucosidase successfully converted more than 99.9% of platycoside E and platycodin D3 into PLD under optimal conditions within 2 h. With the presents of 0.08 mM PE and 0.12 mM PD3, the maximum level of PLD was 0.4 mM (Ahn et al., 2018). In the study completed by Kil and his co-workers, PLD was obtained *via* hydrolyzing platycoside E, increasing the total PLD content to 48% (Kil et al., 2019). It is significant for reducing production costs and expanding applications to develop new processes.

Furthermore, more and more saponins have been utilized as excipients for the preparation of nano-delivery systems. It is well known that low water solubility hinders the bioavailability of active drugs. Previous studies have demonstrated that the nano-delivery system composed of amphiphilic substances can enhance the water solubility and bioavailability of drugs, as well as amphiphilic saponins, are one of them. For example, disodium glycyrrhizin loaded 7-ethyl-10-hydroxycamptothecin (Sun et al., 2019) and Chrysomycin A (Xu et al., 2021) were self-formed into nano-micelles. The pharmacokinetic and distribution properties were significantly boosted compared to the untreated mixture group and the free drug group. Owing to the molecular structural similarities with glycyrrhetinic acid, PLD could be a solubilizing agent or a nano-formulation material. Currently, the surface activity of PLD has received more attention, and some scholars have started to study its solubilizing activity and preparations (Ding et al., 2015). Besides, oleanane-type saponins are supposed to be adjuvants in vaccines (Masullo et al., 2017). Sun et al. found that PLD could increase the antigen-specific IgG, IgG1, IgG2A, and IgG2B antibody titers. Therefore, PLD has the potential to be an immunologic adjuvant (Sun et al., 2011). It is crucial that the quality of PG must be seriously considered due to the high demand in the market. Better regulation of the market will ensure not only the source and yield of PLD, but also the efficacy of the formulation. Moreover, based on cellular and animal models, most of the studies on PLD are pre-clinical. Its pharmacological activities, pharmacokinetics, and preparations need to be confirmed by further studies. Obviously, the clinical translation of PLD still has a long way to go. In the future, based on adequate pre-clinical research, a series of research on its clinical application should be strengthened to expand the scope of clinical applications from traditional applications to new fields such as anti-cancer, anti-cancer, anti-virus, and as adjuvant materials.

## Glossary

Ang II: angiotensin II
AUC: area under the plasma concentration curve
BAT: brown adipose tissue
BDL: bile duct ligation
BMMs: bone marrow macrophages
BMI: body-mass index
BUN: blood urea nitrogen
Cmax: peak plasma concentration
CAT: catalase
CCND: cyclin D
CK: creatinine kinase
CRE: creatinine
CVT: cerebral venous thrombosis
DAA: direct-acting anti-viral
EC50: 50% effective concentrations
FPE: fermented PG extracts
GMB: glioblastoma multiforme
GPx: glutathione peroxidase
GSH: glutathione
HCC: hepatocellular carcinoma
HCV: Hepatitis C virus
HFD: high-fat diet
HOS: hypo-osmotic swelling
IC50: half-maximal inhibitory concentrations
KLF: Kruppel-like factor
LDH: lactate dehydrogenase
L-DLC: low-density lipoprotein cholesterol
LDLR: low-density lipoprotein receptor
MDA: malondialdehyde
MMP: matrix metalloproteinase
NO: nitric oxide
PCG1α: Peroxisome proliferator-activated receptor γ coactivator 1α
PD-L1: Programmed death-ligand 1
PG: Platycodon grandiflorus A.DC.
PLD: Platycodin D
PPAR: Peroxisome proliferator-activated receptor
PRRSV: Type 2 Porcine Reproductive and Respiratory Syndrome Virus
ROS: superoxide dismutase
SOD: superoxide dismutase
TC: total cholesterol
TG: triglyceride
Tmax: peak plasma concentration
T1/2: half live
TME: tumor microenvironment
TMPRSS2: transmembrane protease serine 2
TOSC: total oxidant-scavenging capacity
UCP1: uncoupled protein 1
4-HNE: 4-hydroxynonenal

## References

[B1] Ahn H. J. You H. J. Park M. S. Johnston T. V. Ku S. Ji G. E. (2018). Biocatalysis of platycoside E and platycodin D3 using fungal extracellular beta-glucosidase responsible for rapid platycodin D production. Int. J. Mol. Sci. 19, 2671. 10.3390/ijms19092671 30205574 PMC6163259

[B2] Ahn Y. M. Kim S. K. Kang J. S. Lee B. C. (2012). Platycodon grandiflorum modifies adipokines and the glucose uptake in high-fat diet in mice and L6 muscle cells. J. Pharm. Pharmacol. 64, 697–704. 10.1111/j.2042-7158.2012.01455.x 22471365

[B3] Bailly C. Vergoten G. (2020). Proposed mechanisms for the extracellular release of PD-L1 by the anticancer saponin platycodin D. Int. Immunopharmacol. 85, 106675. 10.1016/j.intimp.2020.106675 32531711

[B4] Baldini T. Asioli G. M. Romoli M. Carvalho Dias M. Schulte E. C. Hauer L. (2021). Cerebral venous thrombosis and severe acute respiratory syndrome coronavirus-2 infection: A systematic review and meta-analysis. Eur. J. Neurol. 28, 3478–3490. 10.1111/ene.14727 33426733 PMC8014715

[B5] Blach S. Zeuzem S. Manns M. Altraif I. Duberg A. S. Muljono D. H. (2017). Global prevalence and genotype distribution of hepatitis C virus infection in 2015: A modelling study. Lancet Gastroenterol. Hepatol. 2, 161–176. 10.1016/S2468-1253(16)30181-9 28404132

[B6] Ceña-Diez R. Martin-Moreno A. de la Mata F. J. Gómez-Ramirez R. Muñoz E. Ardoy M. (2019). <p&gt;G1-S4 or G2-S16 carbosilan dendrimer in combination with Platycodin D as a promising vaginal microbicide candidate with contraceptive activity&lt;/p&gt;. Int. J. Nanomedicine 14, 2371–2381. 10.2147/ijn.s188495 31040662 PMC6452809

[B7] Chen S. Wang Q. Ming S. Zheng H. Hua B. Yang H. (2022). Platycodin D induces apoptosis through JNK1/AP-1/PUMA pathway in non-small cell lung cancer cells: A new mechanism for an old compound. Front. Pharmacol. 13. 10.3389/fphar.2022.1045375 PMC9723146 36483740

[B8] Chen T. Gao J. Xiang P. Chen Y. Ji J. Xie P. (2015). Protective effect of platycodin D on liver injury in alloxan-induced diabetic mice via regulation of Treg/Th17 balance. Int. Immunopharmacol. 26, 338–348. 10.1016/j.intimp.2015.04.001 25887267

[B9] Choi J. H. Han Y. Kim Y. A. Jin S. W. Lee G. H. Jeong H. M. (2017). Platycodin D inhibits osteoclastogenesis by repressing the NFATc1 and MAPK signaling pathway. J. Cell. Biochem. 118, 860–868. 10.1002/jcb.25763 27739107

[B10] Choi J. H. Jin S. W. Han E. H. Park B. H. Kim H. G. Khanal T. (2014). Platycodon grandiflorum root-derived saponins attenuate atopic dermatitis-like skin lesions via suppression of NF-κB and STAT1 and activation of Nrf2/ARE-mediated heme oxygenase-1. Phytomedicine 21, 1053–1061. 10.1016/j.phymed.2014.04.011 24854572

[B11] Choi Y. H. (2022). Induction of ROS-dependent apoptotic cell death by platycodin D in human prostate cancer PC3 cells. J. Men's Health 18, 83. 10.31083/jomh.2021.132

[B12] Choi Y. J. Lee S. J. Kim H. I. Lee H. J. Kang S. J. Kim T. Y. (2020). Platycodin D enhances LDLR expression and LDL uptake via down-regulation of IDOL mRNA in hepatic cells. Sci. Rep. 10, 19834. 10.1038/s41598-020-76224-w 33199761 PMC7670405

[B13] Chun J. Joo E. J. Kang M. Kim Y. S. (2013). Platycodin D induces anoikis and caspase-mediated apoptosis via p38 MAPK in AGS human gastric cancer cells. J. Cell. Biochem. 114, 456–470. 10.1002/jcb.24386 22961809

[B14] Chun J. Kim Y. S. (2013). Platycodin D inhibits migration, invasion, and growth of MDA-MB-231 human breast cancer cells via suppression of EGFR-mediated Akt and MAPK pathways. Chem.-Biol. Interact. 205, 212–221. 10.1016/j.cbi.2013.07.002 23867902

[B15] Collaborators G. B. D. O. Afshin A. Forouzanfar M. H. Reitsma M. B. Sur P. Estep K. (2017). Health effects of overweight and obesity in 195 countries over 25 years. N. Engl. J. Med. 377, 13–27. 10.1056/NEJMoa1614362 28604169 PMC5477817

[B16] Collin F. (2019). Chemical basis of reactive oxygen species reactivity and involvement in neurodegenerative diseases. Int. J. Mol. Sci. 20, 2407. 10.3390/ijms20102407 31096608 PMC6566277

[B17] Committee N. P. (2020). Pharmacopoeia of the People's Republic of China. Beijing: China Medical Science and Technology Press.

[B18] Conti F. Buonfiglioli F. Scuteri A. Crespi C. Bolondi L. Caraceni P. (2016). Early occurrence and recurrence of hepatocellular carcinoma in HCV-related cirrhosis treated with direct-acting antivirals. J. Hepatol. 65, 727–733. 10.1016/j.jhep.2016.06.015 27349488

[B19] Deng S. T. (1998). Treatment of 48 cases of acute and chronic pharyngitis with Jiegeng Gancao decoction. Shaanxi J. Tradit. Chin. Med., 200.

[B20] Deng Y. Ren H. Ye X. Xia L. Liu M. Liu Y. (2020). Integrated phytochemical analysis based on UPLC-Q-TOF-MS/MS, network pharmacology, and experiment verification to explore the potential mechanism of Platycodon grandiflorum for chronic bronchitis. Front. Pharmacol. 11, 564131. 10.3389/fphar.2020.564131 33013400 PMC7506058

[B21] Ding H. Yin Q. Wan G. Dai X. Shi X. Qiao Y. (2015). Solubilization of menthol by platycodin D in aqueous solution: An integrated study of classical experiments and dissipative particle dynamics simulation. Int. J. Pharm. 480, 143–151. 10.1016/j.ijpharm.2015.01.033 25615986

[B22] Docherty A. B. Harrison E. M. Green C. A. Hardwick H. E. Pius R. Norman L. (2020). Features of 20 133 UK patients in hospital with Covid-19 using the ISARIC WHO clinical characterisation protocol: Prospective observational cohort study. BMJ 369, m1985. 10.1136/bmj.m1985 32444460 PMC7243036

[B23] Fan M.-l. Wei K. Wei X.-m. Zhang J.-j. Hou J.-g. Shen Q. (2022). Platycodin D restores the intestinal mechanicalbarrier by reducing endoplasmic reticulum stress-mediated apoptosis. J. Funct. Foods 99, 105336. 10.1016/j.jff.2022.105336

[B24] Fu Y. Xin Z. Liu B. Wang J. Wang J. Zhang X. (2017). Platycodin D inhibits inflammatory response in LPS-stimulated primary rat microglia cells through activating lxrα-ABCA1 signaling pathway. Front. Immunol. 8, 1929. 10.3389/fimmu.2017.01929 29375565 PMC5767310

[B25] Fu Y. Xin Z. Liu B. Wang J. Wang J. Zhang X. (2017). Platycodin D inhibits inflammatory response in LPS-stimulated primary rat microglia cells through activating lxrα-ABCA1 signaling pathway. Front. Immunol. 8, 1929. 10.3389/fimmu.2017.01929 29375565 PMC5767310

[B26] Gao W. Guo Y. Yang H. (2017). Platycodin D protects against cigarette smoke-induced lung inflammation in mice. Int. Immunopharmacol. 47, 53–58. 10.1016/j.intimp.2017.03.009 28363109

[B27] Gilabert-Oriol R. Mergel K. Thakur M. von Mallinckrodt B. Melzig M. F. Fuchs H. (2013). Real-time analysis of membrane permeabilizing effects of oleanane saponins. Bioorg. Med. Chem. 21, 2387–2395. 10.1016/j.bmc.2013.01.061 23454223

[B28] Greten F. R. Grivennikov S. I. (2019). Inflammation and cancer: Triggers, mechanisms, and consequences. Immunity 51, 27–41. 10.1016/j.immuni.2019.06.025 31315034 PMC6831096

[B29] Guo R. Meng Q. Wang B. Li F. (2021). Anti-inflammatory effects of Platycodin D on dextran sulfate sodium (DSS) induced colitis and *E. coli* Lipopolysaccharide (LPS) induced inflammation. Int. Immunopharmacol. 94, 107474. 10.1016/j.intimp.2021.107474 33611056

[B30] Hekman R. M. Hume A. J. Goel R. K. Abo K. M. Huang J. Blum B. C. (2020). Actionable cytopathogenic host responses of human alveolar type 2 cells to SARS-CoV-2. Mol. Cell. 80, 1104–1122.e9. 10.1016/j.molcel.2020.11.028 33259812 PMC7674017

[B31] Hsu W. C. Ramesh S. Shibu M. A. Chen M. C. Wang T. F. Day C. H. (2021). Platycodin D reverses histone deacetylase inhibitor resistance in hepatocellular carcinoma cells by repressing ERK1/2-mediated cofilin-1 phosphorylation. Phytomedicine 82, 153442. 10.1016/j.phymed.2020.153442 33412494

[B32] Hu J. N. Leng J. Shen Q. Liu Y. Li X. D. Wang S. H. (2021). Platycodin D suppresses cisplatin-induced cytotoxicity by suppressing ROS-mediated oxidative damage, apoptosis, and inflammation in HEK-293 cells. J. Biochem. Mol. Toxicol. 35, e22624. 10.1002/jbt.22624 32881195

[B33] Hu X. Fu Y. Lu X. Zhang Z. Zhang W. Cao Y. (2016). Protective effects of platycodin D on lipopolysaccharide-induced acute lung injury by activating lxrα-ABCA1 signaling pathway. Front. Immunol. 7, 644. 10.3389/fimmu.2016.00644 28096801 PMC5206804

[B34] Huang G. R. (1993). Treatment of 187 cases of chronic pharyngitis with Jiawei Jiegeng decoction. Shaanxi J. Tradit. Chin. Med., 32.

[B35] Huang J. Chen G. Wang J. Liu S. Su J. (2022). Platycodin D regulates high glucose-induced ferroptosis of HK-2 cells through glutathione peroxidase 4 (GPX4). Bioengineered 13, 6627–6637. 10.1080/21655979.2022.2045834 35226829 PMC8973889

[B36] Huang M. Y. Jiang X. M. Xu Y. L. Yuan L. W. Chen Y. C. Cui G. (2019). Platycodin D triggers the extracellular release of programed death Ligand-1 in lung cancer cells. Food Chem. Toxicol. 131, 110537. 10.1016/j.fct.2019.05.045 31150782

[B37] Hwang Y. P. Choi J. H. Kim H. G. Khanal T. Song G. Y. Nam M. S. (2013). Saponins, especially platycodin D, from Platycodon grandiflorum modulate hepatic lipogenesis in high-fat diet-fed rats and high glucose-exposed HepG2 cells. Toxicol. Appl. Pharmacol. 267, 174–183. 10.1016/j.taap.2013.01.001 23319015

[B38] Jang Y. Kim T. Y. Jeon S. Lim H. Lee J. Kim S. (2022). Synthesis and structure–activity relationship study of saponin-based membrane fusion inhibitors against SARS-CoV-2. Bioorg. Chem. 127, 105985. 10.1016/j.bioorg.2022.105985 35809512 PMC9233891

[B39] Jeon S. J. Lee H. J. Lee H. E. Park S. J. Gwon Y. Kim H. (2017). Oleanolic acid ameliorates cognitive dysfunction caused by cholinergic blockade via TrkB-dependent BDNF signaling. Neuropharmacology 113, 100–109. 10.1016/j.neuropharm.2016.07.029 27470063

[B40] Jin L. Han B. Siegel E. Cui Y. Giuliano A. Cui X. (2018). Breast cancer lung metastasis: Molecular biology and therapeutic implications. Cancer Biol. Ther. 19, 858–868. 10.1080/15384047.2018.1456599 29580128 PMC6300341

[B41] Jin Y.-Y. Li Y.-J. Ge D.-S. Zhu L.-L. Wang Y.-Y. Luo J. (2021). Combination of Platycodin D with docetaxel synergistically suppressed cell growth in DU-145 by enhancing apoptosis and alleviating autophagy. Eur. J. Integr. Med. 42, 101302. 10.1016/j.eujim.2021.101302

[B42] Jo H. Oh J. H. Park D. W. Lee C. Min C. K. (2020). Oleanolic acid 3-acetate, a minor element of ginsenosides, induces apoptotic cell death in ovarian carcinoma and endometrial carcinoma cells via the involvement of a reactive oxygen species-independent mitochondrial pathway. J. Ginseng Res. 44, 96–104. 10.1016/j.jgr.2018.09.003 32095097 PMC7033343

[B43] Khandia R. Singhal S. Alqahtani T. Kamal M. A. El-Shall N. A. Nainu F. (2022). Emergence of SARS-CoV-2 Omicron (B.1.1.529) variant, salient features, high global health concerns and strategies to counter it amid ongoing COVID-19 pandemic. Environ. Res. 209, 112816. 10.1016/j.envres.2022.112816 35093310 PMC8798788

[B44] Kil T. G. Kang S. H. Kim T. H. Shin K. C. Oh D. K. (2019). Enzymatic biotransformation of balloon flower root saponins into bioactive platycodin D by deglucosylation with caldicellulosiruptor bescii beta-glucosidase. Int. J. Mol. Sci. 20, 3854. 10.3390/ijms20163854 31394870 PMC6721153

[B45] Kim H. L. Park J. Jung Y. Ahn K. S. Um J. Y. Platycodin D. (2019). Platycodin D, a novel activator of AMP-activated protein kinase, attenuates obesity in db/db mice via regulation of adipogenesis and thermogenesis. Phytomedicine 52, 254–263. 10.1016/j.phymed.2018.09.227 30599906

[B46] Kim H. L. Park J. Park H. Jung Y. Youn D. H. Kang J. (2015). Platycodon grandiflorum A. De candolle ethanolic extract inhibits adipogenic regulators in 3T3-L1 cells and induces mitochondrial biogenesis in primary Brown preadipocytes. J. Agric. Food Chem. 63, 7721–7730. 10.1021/acs.jafc.5b01908 26244589

[B47] Kim J. I. Jeon S. G. Kim K. A. Kim J. J. Song E. J. Jeon Y. (2017). Platycodon grandiflorus root extract improves learning and memory by enhancing synaptogenesis in mice Hippocampus. Nutrients 9, 794. 10.3390/nu9070794 28737698 PMC5537907

[B48] Kim J. W. Park S. J. Lim J. H. Yang J. W. Shin J. C. Lee S. W. (2013). Triterpenoid saponins isolated from Platycodon grandiflorum inhibit hepatitis C virus replication. Evid. Based Complement. Altern. Med. 2013, 560417, 10.1155/2013/560417 PMC3893781 24489585

[B49] Kim T. W. Lee H. K. Song I. B. Lim J. H. Cho E. S. Son H. Y. (2013). Platycodin D attenuates bile duct ligation-induced hepatic injury and fibrosis in mice. Food & Chem. Toxicol. 51, 364–369. 10.1016/j.fct.2012.10.017 23116642

[B50] Kim T. W. Song I. B. Lee H. K. Lim J. H. Cho E. S. Son H. Y. (2012). Platycodin D, a triterpenoid sapoinin from Platycodon grandiflorum, ameliorates cisplatin-induced nephrotoxicity in mice. Food Chem. Toxicol. 50, 4254–4259. 10.1016/j.fct.2012.05.022 22617354

[B51] Kim T. Y. Jeon S. Jang Y. Gotina L. Won J. Ju Y. H. (2021). Platycodin D, a natural component of Platycodon grandiflorum, prevents both lysosome- and TMPRSS2-driven SARS-CoV-2 infection by hindering membrane fusion. Exp. Mol. Med. 53, 956–972. 10.1038/s12276-021-00624-9 34035463 PMC8143993

[B52] Kim Y. A. Oh S. H. Lee G. H. Hoa P. T. Jin S. W. Chung Y. C. (2018). Platycodon grandiflorum-derived saponin attenuates the eccentric exercise-induced muscle damage. Food Chem. Toxicol. 112, 150–156. 10.1016/j.fct.2017.12.045 29287792

[B53] Kubatka P. Mazurakova A. Samec M. Koklesova L. Zhai K. Al-Ishaq R. (2021). Flavonoids against non-physiologic inflammation attributed to cancer initiation, development, and progression-3PM pathways. EPMA J. 12, 559–587. 10.1007/s13167-021-00257-y 34950252 PMC8648878

[B54] Kwon M. Ji H. K. Goo S. H. Nam S. J. Kang Y. J. Lee E. (2017). Involvement of intestinal efflux and metabolic instability in the pharmacokinetics of platycodin D in rats. Drug Metab. Pharmacokinet. 32, 248–254. 10.1016/j.dmpk.2017.05.005 28743418

[B55] Kwon O. G. Ku S. K. An H. D. Lee Y. J. (2014). The effects of platycodin D, a saponin purified from platycodi radix, on collagen-induced DBA/1J mouse rheumatoid arthritis. Evid. Based Complement. Altern. Med. 2014, 954508. 10.1155/2014/954508 PMC3913383 24511322

[B56] Lee E. J. Kang M. Kim Y. S. (2012). Platycodin D inhibits lipogenesis through AMPKα-PPARγ2 in 3T3-L1 cells and modulates fat accumulation in obese mice. Planta Med. 78, 1536–1542. 10.1055/s-0032-1315147 22872592

[B57] Lee H. Bae S. Kim Y. S. Yoon Y. (2011). WNT/β-catenin pathway mediates the anti-adipogenic effect of platycodin D, a natural compound found in Platycodon grandiflorum. Life Sci. 89, 388–394. 10.1016/j.lfs.2011.07.006 21798269

[B58] Lee H. J. Lee S. Y. Jeon B. K. Lee J. W. Lee C. J. Lee M. N. (2010). Effect of platycodin D on airway MUC5AC mucin production and gene expression induced by growth factor and proinflammatory factor. Biomol. Ther. 18, 294–299. 10.4062/biomolther.2010.18.3.294

[B59] Lee H. Kang R. Kim Y. S. Chung S. I. Yoon Y. (2010). Platycodin D inhibits adipogenesis of 3T3-L1 cells by modulating Kruppel-like factor 2 and peroxisome proliferator-activated receptor gamma. Phytother. Res. 24 (2), S161–S167. 10.1002/ptr.3054 20024897

[B60] Lee S. Han E. H. Lim M. K. Lee S. H. Yu H. J. Lim Y. H. (2020). Fermented Platycodon grandiflorum extracts relieve airway inflammation and cough reflex sensitivity *in vivo* . J. Med. Food 23, 1060–1069. 10.1089/jmf.2019.4595 32758004

[B61] Lee S. J. Choi Y.-J. Kim H. I. Moon H. E. Paek S. H. Kim T. Y. (2022). Platycodin D inhibits autophagy and increases glioblastoma cell death via LDLR upregulation. Mol. Oncol. 16, 250–268. 10.1002/1878-0261.12966 33931944 PMC8732342

[B62] Lee S. J. Choi Y. J. Kim H. I. Moon H. E. Paek S. H. Kim T. Y. (2021). Platycodin D inhibits autophagy and increases glioblastoma cell death via LDLR upregulation. Mol. Oncol. 16, 250–268. 10.1002/1878-0261.12966 33931944 PMC8732342

[B63] Lee S. K. Park K. K. Kim H. J. Kim K. R. Kang E. J. Kim Y. L. (2015). Platycodin D blocks breast cancer-induced bone destruction by inhibiting osteoclastogenesis and the growth of breast cancer cells. Cell. Physiol. Biochem. 36, 1809–1820. 10.1159/000430152 26184636

[B64] Lei J. Zhao J. Cao X.-W. Wang F.-J. Wang F. J. (2022). In addition to its endosomal escape effect, platycodin D also synergizes with ribosomal inactivation protein to induce apoptosis in hepatoma cells through AKT and MAPK signaling pathways. Chemico-Biological Interact. 364, 110058. 10.1016/j.cbi.2022.110058 35872048

[B65] Li J. Ma A. Lan W. Liu Q. (2022). Platycodon D-induced A549 cell apoptosis through RRM1-regulated p53/VEGF/MMP2 pathway. Anticancer Agents Med. Chem. 22, 2458–2467. 10.2174/1871520622666220128095355 35088678

[B66] Li S. Zhang Y. Guan Z. Li H. Ye M. Chen X. (2020). SARS-CoV-2 triggers inflammatory responses and cell death through caspase-8 activation. Signal Transduct. Target Ther. 5, 235. 10.1038/s41392-020-00334-0 33037188 PMC7545816

[B67] Li T. Chen X. Chen X. Ma D. L. Leung C. H. Lu J. J. (2016). Platycodin D potentiates proliferation inhibition and apoptosis induction upon AKT inhibition via feedback blockade in non-small cell lung cancer cells. Sci. Rep. 6, 37997. 10.1038/srep37997 27897231 PMC5126555

[B68] Li T. Chen X. Dai X. Y. Wei B. Weng Q. J. Chen X. (2017). Novel Hsp90 inhibitor platycodin D disrupts Hsp90/Cdc37 complex and enhances the anticancer effect of mTOR inhibitor. Toxicol. Appl. Pharmacol. 330, 65–73. 10.1016/j.taap.2017.07.006 28711525

[B69] Li T. Tang Z. H. Xu W. S. Wu G. S. Wang Y. F. Chang L. L. (2015). Platycodin D triggers autophagy through activation of extracellular signal-regulated kinase in hepatocellular carcinoma HepG2 cells. Eur. J. Pharmacol. 749, 81–88. 10.1016/j.ejphar.2015.01.003 25592318

[B70] Li T. Xu X. H. Tang Z. H. Wang Y. F. Leung C. H. Ma D. L. (2015). Platycodin D induces apoptosis and triggers ERK- and JNK-mediated autophagy in human hepatocellular carcinoma BEL-7402 cells. Acta Pharmacol. Sin. 36, 1503–1513. 10.1038/aps.2015.99 26592509 PMC4816242

[B71] Li W. Liu Y. Wang Z. Han Y. Tian Y. H. Zhang G. S. (2015). Platycodin D isolated from the aerial parts of Platycodon grandiflorum protects alcohol-induced liver injury in mice. Food Funct. 6, 1418–1427. 10.1039/c5fo00094g 25927324

[B72] Lin H. Wang X. Liu M. Huang M. Shen Z. Feng J. (2021). Exploring the treatment of COVID-19 with Yinqiao powder based on network pharmacology. Phytother. Res. 35, 2651–2664. 10.1002/ptr.7012 33452734 PMC8013442

[B73] Lin Y. C. Lin Y. C. Kuo W. W. Shen C. Y. Chen Y. F. Lin Y. M. (2017). Platycodin D (PD) attenuates myocardial apoptosis mediated by JNK-SIRT1-HSF1-IGF-IIR-Caspase-3 signaling in hypertensive conditions. J. Funct. Foods 34, 59–67. 10.1016/j.jff.2017.04.008

[B74] Lin Y. C. Lin Y. C. Kuo W. W. Shen C. Y. Cheng Y. C. Lin Y. M. (2018). Platycodin D reverses pathological cardiac hypertrophy and fibrosis in spontaneously hypertensive rats. Am. J. Chin. Med. 46, 537–549. 10.1142/S0192415X18500271 29595072

[B75] Liu B. Huang Y. Liu Z. Li D. Dao J. (2022). Platycodin D alleviates high-glucose-aggravated inflammatory responses in oral mucosal cells by PI3K/mTOR pathway. Coatings 12, 444. 10.3390/coatings12040444

[B76] Liu T. J. Wen S. M. (1995). Experienced prescriptions for children's cough. Hunan J. Tradit. Chin. Med., 24.

[B77] Liu T. Zhang L. Y. Joo D. Sun S. C. (2017). Signal Transduction Targeted Ther, 2.NF-kappa B signaling in inflammation 10.1038/sigtrans.2017.23 PMC5661633 29158945

[B78] Liu Y. M. Cong S. Cheng Z. Hu Y. X. Lei Y. Zhu L. L. (2020). Platycodin D alleviates liver fibrosis and activation of hepatic stellate cells by regulating JNK/c-JUN signal pathway. Eur. J. Pharmacol. 876, 172946. 10.1016/j.ejphar.2020.172946 31996320

[B79] Liu Y. Tian S. Yi B. Feng Z. Chu T. Liu J. (2022). Platycodin D sensitizes KRAS-mutant colorectal cancer cells to cetuximab by inhibiting the PI3K/Akt signaling pathway. Front. Oncol. 12, 1046143. 10.3389/fonc.2022.1046143 36387129 PMC9646952

[B80] Lu R. J. Zhao X. Li J. Niu P. H. Yang B. Wu H. L. (2020). Genomic characterisation and epidemiology of 2019 novel coronavirus: Implications for virus origins and receptor binding. Lancet 395, 565–574. 10.1016/S0140-6736(20)30251-8 32007145 PMC7159086

[B81] Lu Z. Song W. Zhang Y. Wu C. Zhu M. Wang H. (2021). Combined anti-cancer effects of platycodin D and sorafenib on androgen-independent and PTEN-deficient prostate cancer. Front. Oncol. 11, 648985. 10.3389/fonc.2021.648985 34026624 PMC8138035

[B82] Lu Z. Wang L. Zhou R. Qiu Y. Yang L. Zhang C. (2013). Evaluation of the spermicidal and contraceptive activity of Platycodin D, a Saponin from Platycodon grandiflorum. PLoS One 8, e82068. 10.1371/journal.pone.0082068 24303079 PMC3841115

[B83] Luan X. Gao Y. G. Guan Y. Y. Xu J. R. Lu Q. Zhao M. (2014). Platycodin D inhibits tumor growth by antiangiogenic activity via blocking VEGFR2-mediated signaling pathway. Toxicol. Appl. Pharmacol. 281, 118–124. 10.1016/j.taap.2014.09.009 25250884

[B84] Luo Q. Wei G. Wu X. Tang K. Xu M. Wu Y. (2018). Platycodin D inhibits platelet function and thrombus formation through inducing internalization of platelet glycoprotein receptors. J. Transl. Med. 16, 311. 10.1186/s12967-018-1688-z 30442147 PMC6238268

[B85] Mao Y. Peng L. Kang A. Xie T. Xu J. Shen C. (2017). Influence of Jiegeng on pharmacokinetic properties of flavonoids and saponins in Gancao. Molecules 22, 1587. 10.3390/molecules22101587 28934158 PMC6151572

[B86] McFadyen J. D. Stevens H. Peter K. (2020). The emerging threat of (Micro)Thrombosis in COVID-19 and its therapeutic implications. Circ. Res. 127, 571–587. 10.1161/CIRCRESAHA.120.317447 32586214 PMC7386875

[B87] Meng Y. L. Wang W. M. Lv D. D. An Q. X. Lu W. H. Wang X. (2017). The effect of Platycodin D on the expression of cytoadherence proteins P1 and P30 in Mycoplasma pneumoniae models. Environ. Toxicol. Pharmacol. 49, 188–193. 10.1016/j.etap.2017.01.001 28073091

[B88] Meng Y. Yang Y. Lu W. Wang Y. Qian F. Wang X. (2014). The inhibition of Platycodin D on Mycoplasma pneumoniae proliferation and its effect on promoting cell growth after anti-Mycoplasma pneumoniae treatment. Front. Cell. Infect. Microbiol. 4, 192. 10.3389/fcimb.2014.00192 25629010 PMC4292783

[B89] Masullo M. Pizza C. Piacente S., Oleanane derivatives for pharmaceutical use: A patent review (2000-2016). Expert Opin. Ther. Pat. 27 (2017) 237–255. 10.1080/13543776.2017.1253680 27782764

[B90] Mushenkova N. V. Bezsonov E. E. Orekhova V. A. Popkova T. V. Starodubova A. V. Orekhov A. N. (2021). Recognition of oxidized lipids by macrophages and its role in atherosclerosis development. Biomedicines 9, 915. 10.3390/biomedicines9080915 34440119 PMC8389651

[B91] Park C. Cha H. J. Lee H. Jeong J. W. Han M. Song K. S. (2022). Induction of apoptosis through inactivation of ROS-dependent PI3K/Akt signaling pathway by platycodin D in human bladder urothelial carcinoma cells. Gen. Physiol. Biophys. 41, 263–274. 10.4149/gpb_2022013 35938960

[B92] Pawlotsky J. M. (2016). Hepatitis C virus resistance to direct-acting antiviral drugs in interferon-free regimens. Gastroenterology 151, 70–86. 10.1053/j.gastro.2016.04.003 27080301

[B93] Pedrosa R. Mustafa D. A. Soffietti R. Kros J. M. (2018). Breast cancer brain metastasis: Molecular mechanisms and directions for treatment. Neuro Oncol. 20, 1439–1449. 10.1093/neuonc/noy044 29566179 PMC6176797

[B94] Peng F. Xiao F. Lin L. (2022). Protective effects of platycodin D3 on airway remodeling and inflammation via modulating MAPK/NF-κB signaling pathway in asthma mice. Evid. Based Complement. Altern. Med. 2022, 1–9. 10.1155/2022/1612829 PMC9385299 35990822

[B95] Qiao Y. Zhang L. Hou C. Li F. (2021). Platycodin D protects pancreatic β-cells from STZ-induced oxidative stress and apoptosis. Food Sci. Technol. 42. 10.1590/fst.63521

[B96] Qin H. Du X. Zhang Y. Wang R. (2014). Platycodin D, a triterpenoid saponin from Platycodon grandiflorum, induces G2/M arrest and apoptosis in human hepatoma HepG2 cells by modulating the PI3K/Akt pathway. Tumour Biol. 35, 1267–1274. 10.1007/s13277-013-1169-1 24048756

[B97] Qu Y. Zhou L. Wang C. (2016). Effects of platycodin D on IL-1β-induced inflammatory response in human osteoarthritis chondrocytes. Int. Immunopharmacol. 40, 474–479. 10.1016/j.intimp.2016.09.025 27743553

[B98] Ryu C. S. Kim C. H. Lee S. Y. Lee K. S. Choung K. J. Song G. Y. (2012). Evaluation of the total oxidant scavenging capacity of saponins isolated from Platycodon grandiflorum. Food Chem. 132, 333–337. 10.1016/j.foodchem.2011.10.086 26434298

[B99] Sarikahya N. B. Nalbantsoy A. Top H. Gokturk R. S. Sumbul H. Kirmizigul S. (2018). Immunomodulatory, hemolytic and cytotoxic activity potentials of triterpenoid saponins from eight Cephalaria species. Phytomedicine 38, 135–144. 10.1016/j.phymed.2017.11.009 29425646

[B100] Shan J. J. Zou J. S. Xie T. Kang A. Zhou W. Xu J. Y. (2015). Effects of Gancao on pharmacokinetic profiles of platycodin D and deapio-platycodin D in Jiegeng. J. Ethnopharmacol. 170, 50–56. 10.1016/j.jep.2015.04.056 25980422

[B101] Shen J. Zhu Y. Zhou B. Kong L. Jin Y. Zhang D. (2021). *In vitro* and *in vivo* evaluation of a water-in-oil microemulsion of platycodin D. Arch. Pharm. Weinh. 354, e2000497. 10.1002/ardp.202000497 33844326

[B102] Shen Q. Zhong Y. T. Liu X. X. Hu J. N. Qi S. M. Li K. (2023). Platycodin D ameliorates hyperglycaemia and liver metabolic disturbance in HFD/STZ-induced type 2 diabetic mice. Food Funct. 14, 74–86. 10.1039/d2fo03308a 36504256

[B103] Shi C. Li Q. Zhang X. (2020). Platycodin D protects human fibroblast cells from premature senescence induced by H2O2 through improving mitochondrial biogenesis. Pharmacology 105, 598–608. 10.1159/000505593 32008007

[B104] Shin K.-C. Seo M.-J. Kim D.-W. Yeom S.-J. Kim Y.-S. (2019). Characterization of β-glycosidase from caldicellulosiruptor owensensis and its application in the production of platycodin D from balloon flower leaf. Catalysts 9, 1025. 10.3390/catal9121025

[B105] Son J.-A. Lee S. K. Park J. Jung M. J. An S.-E. Yang H. J. (2022). Platycodin D inhibits vascular endothelial growth factor-induced angiogenesis by blocking the activation of mitogen-activated protein kinases and the production of interleukin-8. Am. J. Chin. Med. 50, 1645–1661. 10.1142/S0192415X22500690 35848124

[B106] Suh Y. Yang J. H. Yoon J. Y. Choi Y. S. (2018). Platycodin D may improve acne and prevent scarring by downregulating SREBP-1 expression via inhibition of IGF-1R/PI3K/Akt pathway and modulating inflammation with an increase in collagen. Ann. Dermatol. 30, 581–587. 10.5021/ad.2018.30.5.581 33911482 PMC7992470

[B107] Sun H. Chen L. Wang J. Wang K. Zhou J. (2011). Structure-function relationship of the saponins from the roots of Platycodon grandiflorum for hemolytic and adjuvant activity. Int. Immunopharmacol. 11, 2047–2056. 10.1016/j.intimp.2011.08.018 21945665

[B108] Sun X. Zhu D. Cai Y. Shi G. Gao M. Zheng M. (2019). One-step mechanochemical preparation and prominent antitumor activity of SN-38 self-micelle solid dispersion. Int. J. Nanomed. 14, 2115–2126. 10.2147/IJN.S193783 PMC6440449 30988612

[B109] Tabas I. Glass C. K. (2013). Anti-inflammatory therapy in chronic disease: Challenges and opportunities. Science 339, 166–172. 10.1126/science.1230720 23307734 PMC3608517

[B110] Tang Z.-H. Li T. Gao H.-W. Sun W. Chen X.-P. Wang Y.-T. (2014). Platycodin D from Platycodonis Radix enhances the anti-proliferative effects of doxorubicin on breast cancer MCF-7 and MDA-MB-231 cells. Chin. Med. 9, 16–17. 10.1186/1749-8546-9-16 24982689 PMC4075934

[B111] Tang Z. Li T. Gao H. Sun W. Chen X. Wang Y. (2014). Platycodin D from Platycodonis Radix enhances the anti-proliferative effects of doxorubicin on breast cancer MCF-7 and MDA-MB-231 cells. Chin. Med. 9, 16. 10.1186/1749-8546-9-16 24982689 PMC4075934

[B112] Tao W. Su Q. Wang H. Guo S. Chen Y. Duan J. (2015). Platycodin D attenuates acute lung injury by suppressing apoptosis and inflammation *in vivo* and *in vitro* . Int. Immunopharmacol. 27, 138–147. 10.1016/j.intimp.2015.05.005 25981110

[B113] Tian L. Y. Yang C. X. Duan J. (2007). Clinical efficacy of the Chinese herb Radix Platycodon in the treatment of silicosis. China Occup. Med., 307.

[B114] Vo N. N. Q. Fukushima E. O. Muranaka T. (2017). Structure and hemolytic activity relationships of triterpenoid saponins and sapogenins. J. Nat. Med. 71, 50–58. 10.1007/s11418-016-1026-9 27491744

[B115] Wang B. Gao Y. Zheng G. Ren X. Sun B. Zhu K. (2016). Platycodin D inhibits interleukin-13-induced the expression of inflammatory cytokines and mucus in nasal epithelial cells. Biomed. Pharmacother. 84, 1108–1112. 10.1016/j.biopha.2016.10.052 27780139

[B116] Wang F. (2011). How to identify and treat chronic pharyngitis by type. J. Tradit. Chin. Med. 52, 65.

[B117] Wang F. (2010). How to identify and treat plum pneumonia by type. J. Tradit. Chin. Med. 51, 391.

[B118] Wang G. Guo H. Wang X. (2019). Platycodin D protects cortical neurons against oxygen-glucose deprivation/reperfusion in neonatal hypoxic-ischemic encephalopathy. J. Cell. Biochem. 120, 14028–14034. 10.1002/jcb.28677 30945345

[B119] Wang H. Zhong W. Zhao J. Zhang H. Zhang Q. Liang Y. (2019). Oleanolic acid inhibits epithelial-mesenchymal transition of hepatocellular carcinoma by promoting iNOS dimerization. Mol. Cancer Ther. 18, 62–74. 10.1158/1535-7163.MCT-18-0448 30297361

[B120] Wang Y. Che J. B. Zhao H. Tang J. Y. Shi G. N. (2018). Platycodin D inhibits oxidative stress and apoptosis in H9c2 cardiomyocytes following hypoxia/reoxygenation injury. Biochem. Biophys. Res. Commun. 503, 3219–3224. 10.1016/j.bbrc.2018.08.129 30146261

[B121] Wang Y. Zhang X. Wei Z. Wang J. Zhang Y. Shi M. (2017). Platycodin D suppressed LPS-induced inflammatory response by activating LXRα in LPS-stimulated primary bovine mammary epithelial cells. Eur. J. Pharmacol. 814, 138–143. 10.1016/j.ejphar.2017.07.037 28736281

[B122] Wen X. Wang J. Fan J. Chu R. Chen Y. Xing Y. (2022). Investigating the protective effects of platycodin D on non-alcoholic fatty liver disease in a palmitic acid-induced *in vitro* model. J. Vis. Exp. 10.3791/64816 36533835

[B123] Wu F. Zhao S. Yu B. Chen Y. M. Wang W. Song Z. G. (2020). A new coronavirus associated with human respiratory disease in China. Nature 579, 265–269. 10.1038/s41586-020-2008-3 32015508 PMC7094943

[B124] Wu K. Xi J. (2020). Mechanochemical-assisted extraction of platycodin D from the roots of Platycodon grandiflorum with solid alkalis. Ind. Crops Prod. 145, 112026. 10.1016/j.indcrop.2019.112026

[B125] Wu Y. Huang D. Wang X. Pei C. Xiao W. Wang F. (2021). Suppression of NLRP3 inflammasome by Platycodin D via the TLR4/MyD88/NF-κB pathway contributes to attenuation of lipopolysaccharide induced acute lung injury in rats. Int. Immunopharmacol. 96, 107621. 10.1016/j.intimp.2021.107621 33872850

[B126] Wynn T. A. Ramalingam T. R. (2012). Mechanisms of fibrosis: Therapeutic translation for fibrotic disease. Nat. Med. 18, 1028–1040. 10.1038/nm.2807 22772564 PMC3405917

[B127] Xie Y. Ye Y. P. Sun H. X. Li D. (2008). Contribution of the glycidic moieties to the haemolytic and adjuvant activity of platycodigenin-type saponins from the root of Platycodon grandiflorum. Vaccine 26, 3452–3460. 10.1016/j.vaccine.2008.04.023 18501482

[B128] Xu C. Sun G. Yuan G. Wang R. Sun X. (2014). Effects of platycodin D on proliferation, apoptosis and PI3K/Akt signal pathway of human glioma U251 cells. Molecules 19, 21411–21423. 10.3390/molecules191221411 25532840 PMC6270900

[B129] Xu C. Sun G. Yuan G. Wang R. Sun X. (2014). Effects of platycodin D on proliferation, apoptosis and PI3K/Akt signal pathway of human glioma U251 cells. Molecules 19, 21411–21423. 10.3390/molecules191221411 25532840 PMC6270900

[B130] Xu J. Zhang Y. F. (2020). Traditional Chinese medicine treatment of COVID-19. Ther. Clin. Pract. 39, 101165. 10.1016/j.ctcp.2020.101165 PMC7118627 32379692

[B131] Xu Z. Zheng S. Gao X. Hong Y. Cai Y. Zhang Q. (2021). Mechanochemical preparation of chrysomycin A self-micelle solid dispersion with improved solubility and enhanced oral bioavailability. J. Nanobiotechnology 19, 164. 10.1186/s12951-021-00911-7 34059070 PMC8166083

[B132] Yang F. S. Gao F. Tan T. H. Xu Y. Cao F. Wang Z. T. (2020). Prescription and medication regularity of traditional Chinese medicine for treating child pneumonia based on data mining. China J. Chin. Mat. Med. 45, 1942–1947. 10.19540/j.cnki.cjcmm.20190902.501 32489081

[B133] Yang X. B. Yu Y. Xu J. Q. Shu H. Q. Xia J. A. Liu H. (2020). Clinical course and outcomes of critically ill patients with SARS-CoV-2 pneumonia in wuhan, China: A single-centered, retrospective, observational study. Lancet Respir. Med. 8, 475–481. 10.1016/s2213-2600(20)30079-5 32105632 PMC7102538

[B134] Ye Y. Han X. Guo B. Sun Z. Liu S. (2013). Combination treatment with platycodin D and osthole inhibits cell proliferation and invasion in mammary carcinoma cell lines. Environ. Toxicol. Pharmacol. 36, 115–124. 10.1016/j.etap.2013.03.012 23603464

[B135] Ye Y. Pei L. Ding J. Wu C. Sun C. Liu S. (2019). Effects of Platycodin D on S100A8/A9-induced inflammatory response in murine mammary carcinoma 4T1 cells. Int. Immunopharmacol. 67, 239–247. 10.1016/j.intimp.2018.12.008 30562685

[B136] Ye Y. Xie Y. Pei L. Jiang Z. Wu C. Liu S. (2023). Platycodin D induces neutrophil apoptosis by downregulating PD-L1 expression to inhibit breast cancer pulmonary metastasis. Int. Immunopharmacol. 115, 109733. 10.1016/j.intimp.2023.109733 37724959

[B137] Yin X. Fang X. (2021). The effect of Platycodon grandiflorum and its historical change in the clinical application of Platycodonis radix. Chin. J. Med. Hist. 51, 167–176. 10.3760/cma.j.cn112155-20201222-00201 34645201

[B138] Yu Z. Li Y. Fu R. Xue Y. Zhao D. Han D. (2022). Platycodin D inhibits the proliferation and migration of hypertrophic scar-derived fibroblasts and promotes apoptosis through a caspase-dependent pathway. Arch. Dermatol. Res., 1–11. 10.1007/s00403-022-02513-1 PMC10205854 36526799

[B139] Zeng C. C. Zhang C. Yao J. H. Lai S. H. Han B. J. Li W. (2016). Platycodin D induced apoptosis and autophagy in PC-12 cells through mitochondrial dysfunction pathway. Spectrochim. Acta. A Mol. Biomol. Spectrosc. 168, 199–205. 10.1016/j.saa.2016.06.005 27294548

[B140] Zhang H. Gong X. Peng Y. Saravanan K. M. Bian H. Zhang J. Z. H. (2022). An efficient modern strategy to screen drug candidates targeting RdRp of SARS-CoV-2 with potentially high selectivity and specificity. Front. Chem. 10, 933102. 10.3389/fchem.2022.933102 35903186 PMC9315156

[B141] Zhang J. Song N. Liu Y. Guo J. (2021). Platycodin D inhibits β-amyloid-induced inflammation and oxidative stress in BV-2 cells via suppressing TLR4/NF-κB signaling pathway and activating Nrf2/HO-1 signaling pathway. Neurochem. Res. 46, 638–647. 10.1007/s11064-020-03198-6 33394221

[B142] Zhang L. Wang Y. L. Yang D. W. Zhang C. H. Zhang N. Li M. H. (2015). Platycodon grandiflorus - an Ethnopharmacological, phytochemical and pharmacological review. J. Ethnopharmacol. 164, 147–161. 10.1016/j.jep.2015.01.052 25666431

[B143] Zhang L. Xue S. Ren F. Huang S. Zhou R. Wang Y. (2021). An atherosclerotic plaque-targeted single-chain antibody for MR/NIR-II imaging of atherosclerosis and anti-atherosclerosis therapy. J. Nanobiotechnology 19, 296. 10.1186/s12951-021-01047-4 34583680 PMC8479957

[B144] Zhang M. Du T. Long F. Yang X. Sun Y. Duan M. (2018). Platycodin D suppresses type 2 porcine reproductive and respiratory syndrome virus in primary and established cell lines. Viruses 10, 657. 10.3390/v10110657 30469357 PMC6266211

[B145] Zhang S. Liu Y. Wang X. Yang L. Li H. Wang Y. (2020). SARS-CoV-2 binds platelet ACE2 to enhance thrombosis in COVID-19. J. Hematol. Oncol. 13, 120. 10.1186/s13045-020-00954-7 32887634 PMC7471641

[B146] Zhang T. Yang S. Du J. Jinfu Y. Shumin W. (2015). Platycodin D attenuates airway inflammation in a mouse model of allergic asthma by regulation NF-κB pathway. Inflammation 38, 1221–1228. 10.1007/s10753-014-0089-6 25578175

[B147] Zhao R. Chen M. Jiang Z. Zhao F. Xi B. Zhang X. (2015). Platycodin-D induced autophagy in non-small cell lung cancer cells via PI3K/Akt/mTOR and MAPK signaling pathways. J. Cancer 6, 623–631. 10.7150/jca.11291 26078792 PMC4466411

[B148] Zheng S. Xie Z. Xin Y. Lu W. Yang H. Lu T. (2022). Whole transcriptome analysis identifies platycodin D-mediated RNA regulatory network in non–small-cell lung cancer. Cells 11, 2360. 10.3390/cells11152360 35954204 PMC9367903

[B149] Zhu N. Zhang D. Wang W. Li X. Yang B. Song J. (2020). A novel coronavirus from patients with pneumonia in China, 2019. N. Engl. J. Med. 382, 727–733. 10.1056/NEJMoa2001017 31978945 PMC7092803

